# Design and Optimization of a Silicon-Based Electrokinetic Microchip for Sensitive Detection of Small Extracellular Vesicles

**DOI:** 10.1021/acssensors.4c00110

**Published:** 2024-06-07

**Authors:** Moein Talebian Gevari, Siddharth Sourabh Sahu, Fredrik Stridfeldt, Petra Hååg, Luigi De Petris, Kristina Viktorsson, Rolf Lewensohn, Alessandro Gori, Marina Cretich, Apurba Dev

**Affiliations:** † Division of Solid-State Electronics, Department of Electrical Engineering, Uppsala University, 75 121 Uppsala, Sweden; ‡ Department of Applied Physics, School of Engineering Sciences, KTH Royal Institute of Technology, 10 691 Stockholm, Sweden; § Department of Oncology-Pathology, Karolinska Institutet, 171 64 Solna, Sweden; ∥ Theme Cancer, Medical Unit Head and Neck, Lung, and Skin Tumors, Thoracic Oncology Center, Karolinska University Hospital, 171 64 Solna, Sweden; ⊥ Consiglio Nazionale delle Ricerche, Istituto di Scienze e Tecnologie Chimiche “Giulio Natta” (SCITEC), 20131 Milan, Italy

**Keywords:** microchip biosensor, extracellular vesicles, microfluidics, streaming current, electrokinetic effects

## Abstract

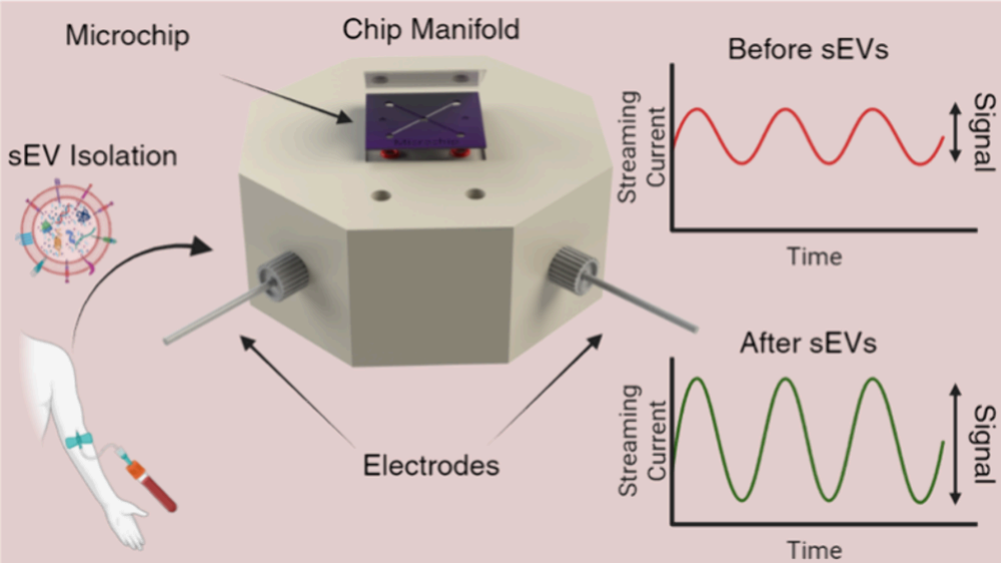

Detection of analytes using streaming current has previously been explored using both experimental approaches and theoretical analyses of such data. However, further developments are needed for establishing a viable microchip that can be exploited to deliver a sensitive, robust, and scalable biosensor device. In this study, we demonstrated the fabrication of such a device on silicon wafer using a scalable silicon microfabrication technology followed by characterization and optimization of this sensor for detection of small extracellular vesicles (sEVs) with sizes in the range of 30 to 200 nm, as determined by nanoparticle tracking analyses. We showed that the sensitivity of the devices, assessed by a common protein–ligand pair and sEVs, significantly outperforms previous approaches using the same principle. Two versions of the microchips, denoted as enclosed and removable-top microchips, were developed and compared, aiming to discern the importance of high-pressure measurement versus easier and better surface preparation capacity. A custom-built chip manifold allowing easy interfacing with standard microfluidic connections was also constructed. By investigating different electrical, fluidic, morphological, and fluorescence measurements, we show that while the enclosed microchip with its robust glass-silicon bonding can withstand higher pressure and thus generate higher streaming current, the removable-top configuration offers several practical benefits, including easy surface preparation, uniform probe conjugation, and improvement in the limit of detection (LoD). We further compared two common surface functionalization strategies and showed that the developed microchip can achieve both high sensitivity for membrane protein profiling and low LoD for detection of sEV detection. At the optimum working condition, we demonstrated that the microchip could detect sEVs reaching an LoD of 10^4^ sEVs/mL (when captured by membrane-sensing peptide (MSP) probes), which is among the lowest in the so far reported microchip-based methods.

During the past years, the principle of electrokinetic biosensing, exploiting streaming current/potential, has been applied in the detection of a wide variety of bioanalytes including proteins,^1−3^ DNA,^4^ and extracellular vesicles (EVs),^5^ thereby demonstrating a potential of the method as a generic biosensor approach. Electrokinetic biosensing relies on the electrostatic and hydrodynamic interaction at the solid–liquid interface inside a microchannel and allows for label-free detection of bioanalytes. A major benefit is its high sensitivity to the surface coverage of an analyte, which previously has been studied with inorganic particles^6^ and, lately, for the determination of bioanalyte concentrations.^7^ Besides, the method offers several practical advantages, such as low sample consumption, simple and inexpensive device architecture, and possibility to integrate with standard microfluidic technologies for sample sorting,^8^ enrichment, and deliveries. These advantages have attracted further interest in the method, aiming for both improved understanding and exploitation of the governing principles. We have recently demonstrated that the surface charge density and the charge contrast between the sensor surface and analytes play a major role, which can be exploited to achieve a better biosensor sensitivity.^9,10^ Furthermore, by designing an appropriate charge-labeled detection probe, we showed the possibility to develop an immunosandwich assay, thereby extending the application of the method also to assessment of complex bioanalytes.^9^ We also reported on a multiplexed detection setup for simultaneous measurement of several bioanalytes.^11^

Clearly, the detection principle has matured in its ability to study bioanalytes, significantly improving in both sensitivity and specificity. A further understanding of how the physical parameters of an analyte, such as its size and charge, influence the sensor response^12^ now provides us new opportunities to design a more sensitive detector. However, the developments so far have mainly been done using nonscalable channel design, e.g., commercial silica capillaries,^10^ which have limited scope to exploit many of the benefits in practical settings. An implementation of the detection principle on microfabricated channels can help further leverage some of the key advantages of the method. This includes the design of shorter and narrower channels to decrease the sample consumption, scalable fabrication, improvement in the quality of surface oxides for a better sensitivity, and integration of multiple channels for increasing the throughput of multiplexing. In addition, such a microfabricated sensor can open new avenues for research, e.g., new material for sensing surface, integration of fluidic actuation, and exploring of the benefit of nanoengineered surface.

In this study, we report on the fabrication and characterization of such a microchip biosensor realized by microfabrication of fluidic channels on a silicon wafer. Two different designs are presented: an enclosed microchip where a glass wafer is anodically bonded with the microfabricated silicon chip and a removable-top configuration where the microchannels are created by mechanically pressing a polydimethylsiloxane (PDMS)-covered glass against the silicon substrate. While both the devices show a linear and reproducible streaming current as a function of the applied pressure, the removable-top chip offers several practical benefits without compromising sensitivity and LoD. The removable-top microchip offers full access to the active surface of the microchannels, which can be used to efficiently functionalize it and enhance the sensitivity of the biosensor. In addition, it can be used for the multiplexed detection of sEVs on one microchip. Using a biotin–streptavidin pair and the EV surface tetraspanins (CD9 and CD81) expressed on small EVs (sEVs) isolated from cell culture media of a nonsmall cell lung cancer (NSCLC) cell line, we then demonstrate that the device can outperform the previous report on LoD using the same detection principle. We also show that surface functionalization strategies can be exploited to further improve the device performance. Thus, we demonstrate that by using a silane-based functionalization strategy, the microchip can achieve an LoD of 10^4^ sEVs/mL when captured by membrane-sensing peptide (MSP) probes.^13−15^ Finally, we use the optimized microchip sensing technique to profile two clinically relevant transmembrane proteins, i.e., CD73 and PD-L1, expressed on sEVs isolated from plasma of an advanced cancer patient at the baseline prior to start of treatment. These developments are expected to take the sensing principle one step closer to clinical applications.

## Materials and Methods

**Figure 1:**
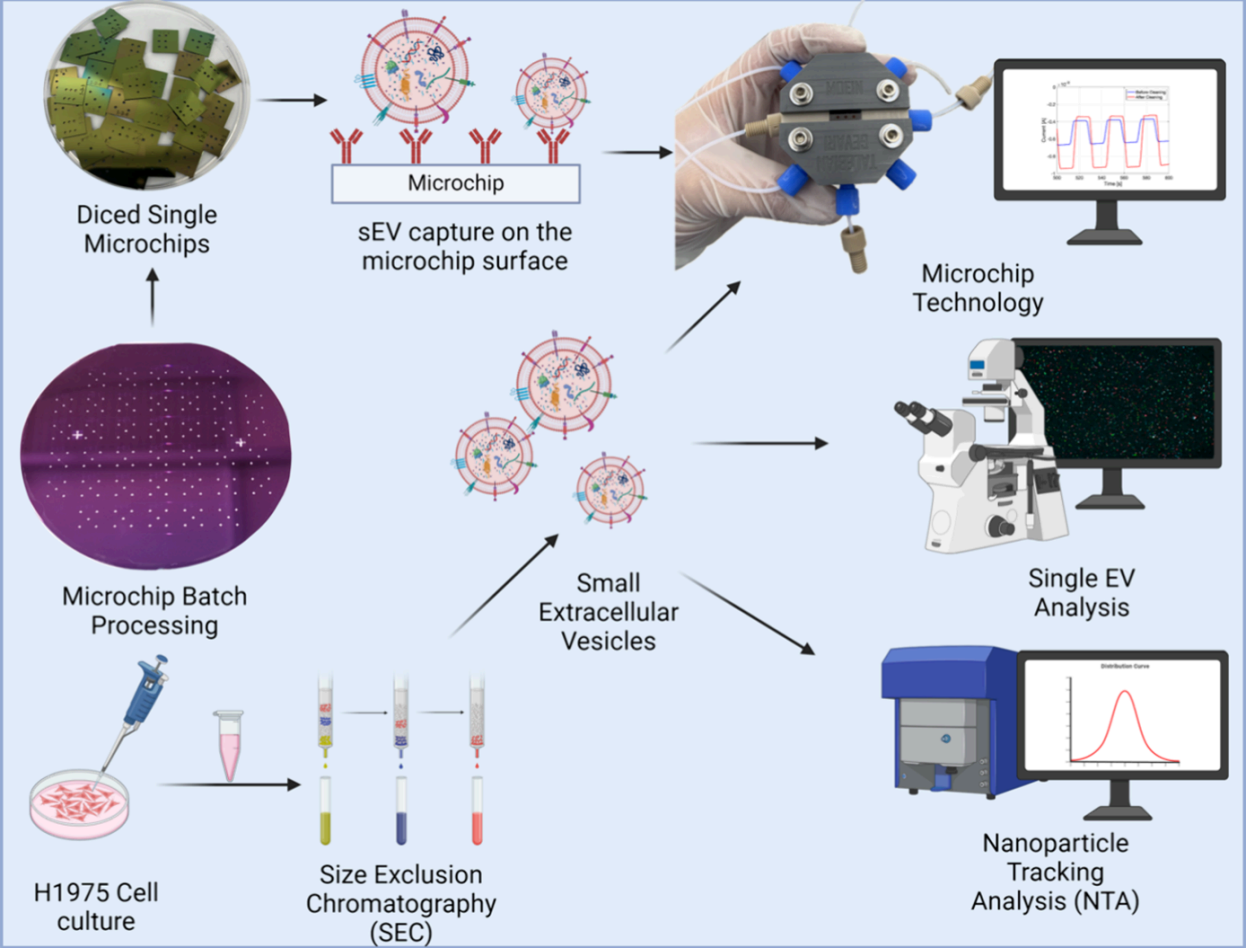
Microchips were batch processed on a 4 inch wafer and diced into small devices for streaming current-based detection of small extracellular vesicles (sEVs) isolated from NSCLC H1975 cell culture media. sEVs were harvested by size exclusion chromatography (SEC). The isolated particles were characterized by a ZetaView nanoparticle tracking analysis system for concentration, size, and zeta potential. The sEVs were further studied using a single-EV platform for their surface protein expression of tetraspanins CD9 and CD81. Created with BioRender.com.

In this study, the microfluidic devices were batch processed on a silicon wafer and then diced into individual sensors chips. Each microchip consisted of four interconnected microchannels of rectangular cross sections, sharing a common inlet port. The surfaces of the microchannels were made of thermally grown silicon dioxide. The morphology and surface roughness of the devices were analyzed by scanning electron microscopy (Zeiss Leo 1530 SEM) and a ZYGO optical profiler (Nexview NX2), respectively. The uniformity of the chemical functionalization was investigated by atomic force microscopy (AFM; JPK NanoWizard 3) and fluorescence analysis (ZEISS Axio Observer 7). The wettability of the surface was analyzed by a custom-made setup consisting of a Dino-Lite AM7115MTF camera, and the images were characterized by an ImageJ contact angle module. After appropriate surface cleaning and chemical functionalization, the detection sensitivity of the devices was tested with streptavidin and sEVs isolated from cell culture media of the NSCLC H1975 cell line as previously described.^16^ A schematic of the workflow used in this study is presented in Figure 1.

### Reagents

For the study, we used pure deionized water (resistivity: 18 MΩ•cm) locally produced. Phosphate-buffered saline (PBS) tablets, avidin from egg white, streptavidin (SA) from *Streptomyces avidinii*, Atto 565-conjugated SA, hydrogen peroxide, and ammonium hydroxide were purchased from Sigma-Aldrich Sweden AB (Stockholm, Sweden). The capturing probes consisting of poly(l-lysine)-*graft*-biotinylated PEG (PLL-*g*-PEG-biotin, referred to as PPB here after) were purchased from Nanosoft Polymers (Winston Salem, North Carolina, USA). Silane-PEG-biotin (referred to as SPB here after) was purchased from Laysan Bio (Arab, Alabama, USA). Biotinylated human anti-CD9 antibody (MEM-61; catalog no. MA1-19485) and biotinylated human anti-CD81 antibody (M38; catalog no. ab239238) were purchased from Thermo Fisher Scientific (Stockholm, Sweden) and Abcam (Cambridge, UK), respectively. Biotinylated human anti-CD73 antibody (AD2; catalog no. 344017) and biotinylated human anti-PD-L1 antibody (B7-H1; catalog no. BAF156) were purchased from Nordic Biosite (Stockholm, Sweden) and Bio-Techne (Dublin, Ireland), respectively.

To minimize the nonspecific interaction of the sEVs with the microchip surface, pluronic (synperonic) F108 was purchased from Sigma-Aldrich Sweden AB (Stockholm, Sweden). For the single-EV platform, anti-CD9 antibody conjugated with VioBlue was bought from Miltenyi Biotec Norden AB (Lund, Sweden) (catalog no. 130-118-809), and anti-CD81-APC (catalog no. A87789) was purchased from Beckman Coulter, USA. All detection antibodies used on the single-EV platform were monoclonal.

### Extracellular Vesicles from Cell Culture Media—Isolation and Characterization

The sEV isolation from the cell culture media of the NSCLC cell line H1975 (ATCC CRL-5908, distributor LGC Standards, Middlesex, UK) in this study followed in principle our previous work.^16^ In short, two steps of centrifugation were performed on the cell culture media. Size exclusion chromatography (SEC) on qEVoriginal Gen 2, 70 nm, was performed to isolate sEVs. Finally, the particle size and charge were characterized by nanoparticle tracking analysis (NTA, ZetaView from Particle Metrix). More details on the sEVs isolation are given in the Supporting Information (Section S1).

### Nonsmall Cell Lung Cancer Plasma Sample—Isolation, Characterization, and Proximity Extension Assay Profiling of Extracellular Vesicles

The plasma sample that was the source of sEVs in this study was obtained from a 59-year-old man, a former smoker, with advanced lung adenocarcinoma (clinical stage T4 N3M1c) treated at the Karolinska University Hospital, Stockholm, Sweden. The sample was collected within a Biobank study approved by the ethical review board (No. 2018/2668-31/1 and 2019-00093). The plasma sample was taken at baseline, prior to the start of treatment with an immune checkpoint inhibitor regimen in combination with chemotherapy. The blood sample in an EDTA tube was centrifuged at 2400*g* for 10 min at room temperature, and the resulting plasma was frozen at −80 °C until sEV isolation. The sEV isolation from plasma was done from 0.35 mL of frozen plasma using SEC on qEVoriginal gen2, 70 nm, columns (Izon Science, Lyon, France) with details presented in the Supporting Information (Section S2).

The two proteins, programmed death-ligand 1 (PD-L1, encoded by the gene CD274, UniProt. no. Q9NZQ7) and cluster of differentiation 73 (CD73) also known as ecto-5′-nucleotidase (encoded by the gene *NT5E*, UniProt no. P21589) were also assessed on a sample of lysed sEVs using proximity extension assay (PEA) (Olink Proteomics AB, Uppsala, Sweden).

The use of the PEA method has previously been described for protein profiling of EVs/exosomes obtained from cancer cell lines and patient plasma.^17,18^ The PEA assay was carried out on sEVs isolated from the same patient plasma but using another isolate with the same particle size and concentration. The PEA was done according to the manufacturer’s instructions by Affinity Proteomics Uppsala, SciLifeLab, Uppsala University, Sweden. For details, see the Supporting Information (Section S2).

### Peptide Design and Synthesis

Synthetic membrane-sensing peptides (MSPs) derived from Bradykinin were used in the study for sEV capture. Unlike antibodies, MSPs show specific affinity for a highly curved lipid membrane, which can be considered a shared “epitope” for nanovesicles, making MSPs agnostic to the relative abundance of the EV surface proteins. The peptide synthesis, e.g., Branched Peptide, followed our previous report.^13^ However, modifications including a short PEG linker and a terminal Biotin handle for surface immobilization was introduced to fit our biosensor. The probe was assembled by stepwise microwave-assisted Fmoc-SPPS on a Biotage Alstra Initiator+ peptide synthesizer, purified by reversed-phase high-performance liquid chromatography (RP-HPLC) and analyzed by electrospray ionization mass spectrometry (ESI-MS), as previously described.^13^

### Sensing Method, Measurement Setup, and the Microchip

The applied sensing principle is described in our previous articles.^5,11,19^ In short, a PBS buffer was pushed through the microchannels under hydraulic pressure to generate a streaming current. A brief theoretical explanation can be found in the Supporting Information (Section S3). The current was then measured by using a pair of platinum (Pt) electrodes connected at both ends of the channel. The experimental setup along with the procedure is shown in Supporting Information (Section S4).

**Figure 2:**
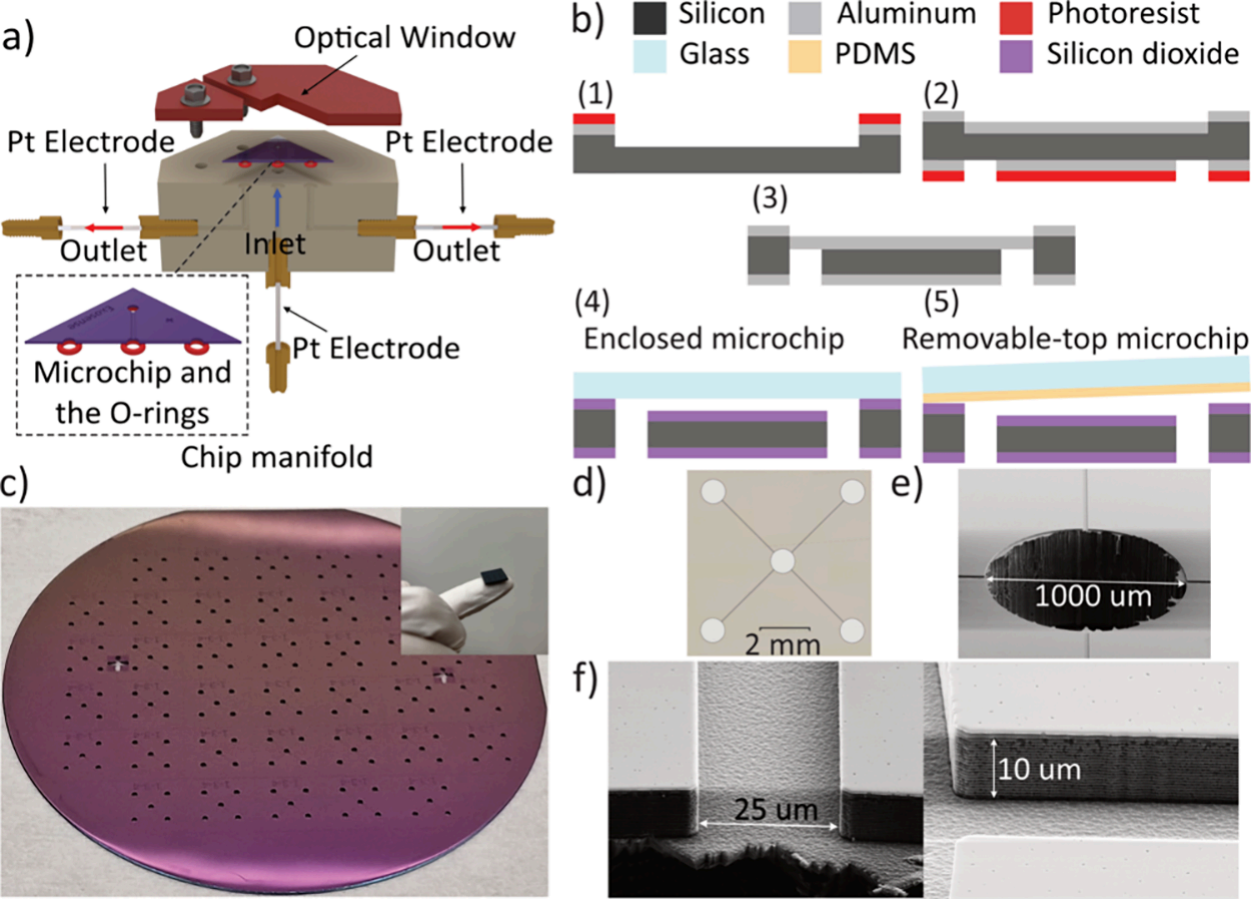
Details of the microchip fabrication and the chip manifold design. (a) Schematic cross-sectional view of the chip manifold and the microchip along with the platinum electrodes and the O-rings. (b) Fabrication process flow schematic: (1) microchannels dry etched into silicon using a sputtered aluminum hard mask, (2) backside optical lithography opening the fluidic ports on the aluminum mask, (3) etch through silicon to create the inlet and outlet ports, (4) thermal oxide growth and anodic bonding with glass for enclosed chip, (5) thermal oxide growth and mechanical bonding with PDMS-covered glass. (c) A fully processed wafer and a single microchip on a fingertip. (d) Optical image of the microchip and scanning electron microscopy image of (e) the inlet port and (f) microchannel cross section and walls.

The centerpiece of the assembled system is a custom-built manifold to mount the microchip. For this purpose, an octagon-shaped PEEK block was machined on the center of which the microchip was placed (Figure 2a and Figure S1). Silicone O-rings were used to ensure a leakproof fluidic interfacing between the manifold and the microchip. Finally, plastic plates were used to sandwich the microchip on the platform. An optical window was designed on the plastic holders to allow for fluorescence microscopy of the microchannel surface. A custom-made PDMS twin reservoir was used on the removable-top microchip to independently functionalize individual channels for the multiplexed measurements (see the Supporting Information (Section S5)). For this purpose, the PDMS twin reservoir was sandwiched on the PPB- or SPB-covered microchips. Thereafter, different biotinylated antibodies were incubated in separate reservoirs. After a thorough washing step by buffer exchange, the removable-top microchip was used for multiplexed detection of the sEVs. Therefore, two subpopulations of the sEVs could be targeted on one microchip. The cross contamination across the reservoirs was tested using FL-tagged SA (see the Supporting Information (Section S5)). The silicon and glass substrates were purchased from MicroChemicals Company (Ulm, Germany). The Sylgard 184 PDMS kit was procured from Ellsworth Adhesives (Stockholm, Sweden).

### Surface Functionalization

Prior to the antibody/receptor immobilization, the surface of the microchannels was cleaned using an RCA1 (5:1:1 DI-Water: H_2_O_2_:NH_4_OH) solution at 88 °C for 20 min. The Supporting Information (Section S6) demonstrates the effect of the cleaning process on the recorded streaming current. After the cleaning process, the microchips were chemically functionalized either by flowing the chemicals through the channels (for enclosed microchips) or by simply incubating the solution (for the removable-top microchips). Two different surface functionalization strategies were followed in this work: (i) a PPB-based protocol and (ii) an SPB-based strategy. For the PPB-based capture, we followed an optimized strategy as reported in our earlier work.^10^ In brief, the surface of the microchannels was first coated with a thin layer of PPB by supplying an aqueous solution (0.1 mg/mL) of PPB for 15 min. For biosensing, the biotinylated anti-CD9 and anti-CD81 antibodies were conjugated to the PPB-coated surface using avidin as a linker molecule. The concentration of the capture antibody was 50 μg/mL in 1× PBS and was immobilized for 60 min. For the SPB-based functionalization strategy, the surface was first treated by 1 mg/mL SPB in 95% ethanol overnight and then washed with 95% ethanol and DI water. After drying, a 0.05 mg/mL solution of SA in 1× PBS was used as the linker between the antibodies and the surface. The antibody immobilization was identical to the step followed in the PPB case. Prior to the sensing measurements, the microchannels were treated with 0.01%w Pluronic (Synperonic) F108 solution for 30 min in order to suppress nonspecific bindings.

### Fluorescence-Based Single Extracellular Vesicle Analysis

For comparison, fluorescence-based single-EV analysis was performed following our earlier report.^20^ For that, a silica coverslip was functionalized by PPB-AV using the same protocol as that for the microchips. Thereafter, the sEVs were incubated on the surface for 1 h and captured electrostatically irrespective of their surface protein expression. The coverslip containing sEVs was then passivated for 30 min using 0.5 mg/mL casein to suppress nonspecific binding of the fluorophore-tagged antibodies. Fluorophore-tagged anti-CD9 and anti-CD81 antibodies were then incubated on the captured sEVs for 1 h followed by washing with PBS to remove unbound antibodies. Fluorescence imaging was done with a Zeiss inverted epifluorescence microscope under LED excitation.

### Microchip Design and Fabrication

For the fabrication, a 100 nm-thick sputtered aluminum layer (using von Ardenne CS 730 S magnetron sputter) was used as a hard mask for etching the silicon substrate. The patterns defining the channels were lithographically generated on the surface using a KarlSüss MA6 lithography machine. The aluminum mask was dry etched followed by a 10 μm-deep dry etching of the silicon substrate (Figure 2b(1)) both using a PLASMA-THERM SLR 790 DRIE machine. Thereafter, aluminum layers were deposited on the back and front sides of the substrate. A backside photolithography followed by a dry etching of the aluminum mask (Figure 2b(2)) and a deep dry etching of the silicon wafer was done (Figure 2b(3)) to form the inlet and outlet ports. Finally, a 300 nm-thick layer of silicon dioxide was thermally grown on the substrate at 1000 °C by using a Koyo Vertical Diffusion Furnace.

To create the enclosed microchips, the substrate was anodically bonded to a borofloat glass wafer (Figure 2b(4)) by using an in-house designed setup. In the case of the removable-top microchips, a glass wafer was bonded to a 100 μm-thick sheet of PDMS by plasma treatment. The PDMS-covered glass was pressed against the microchips on the chip manifold to create a leakproof fluidic path (Figure 2b(5)). More details of the fabrication process flow could be found in the Supporting Information (Section S7). Figure 2c shows a fully processed wafer containing 34 microchips and a single microchip (12 × 12 mm) on a fingertip.

The cross-sectional dimensions of the microchannels are 10 μm × 25 μm and 3 mm in length. The optical images showing four identical microchannels with a common inlet (at the center) and separate outlets as well as scanning electron microscopy images are presented in Figure 2d–f. The surface roughness of the fabricated devices was characterized using the white light interferometry (AMETEK ZYGO Nexview Optical Surface Profiler) method (see the Supporting Information (Section S8)).

## Results

Electrical and fluidic characterizations were performed to find the relative performance and optical working range of the different microchip configurations. The sensing performance of the microchips was then analyzed by comparing their detection sensitivity and LoD for targeting streptavidin and for sEVs isolated by size exclusion chromatography from cell culture media of an NSCLC cell line as well as from plasma of a patient with lung adenocarcinoma.

### Fluidic and Electrical Characterization

**Figure 3:**
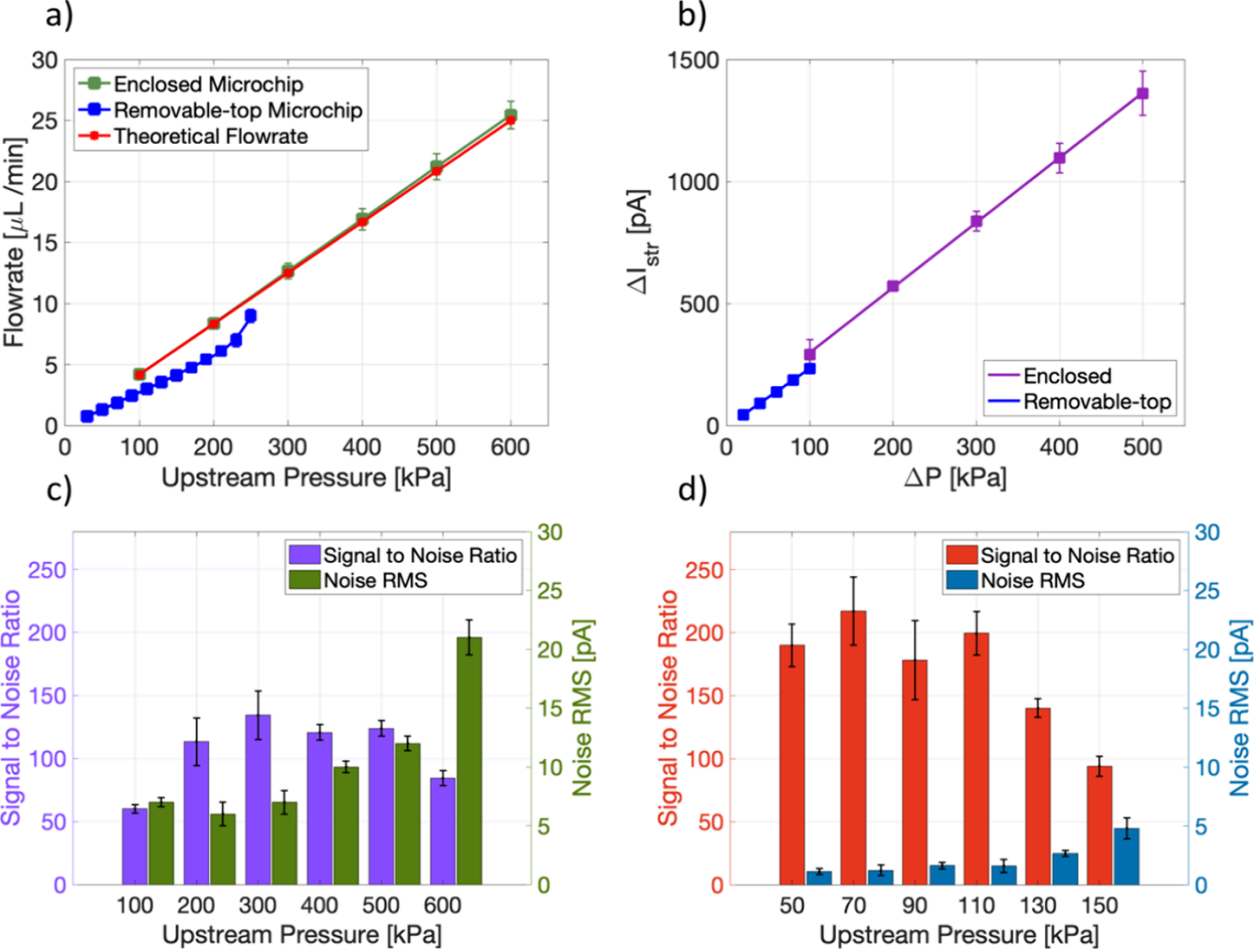
Electrical and fluidic characterization of the microchips. (a) Linear relation between the flow rate and the upstream pressure for both microchips. (b) Linear relation between the streaming current and the upstream pressure for both microchips. (c) Signal-to-noise ratio (SNR) and noise RMS for the enclosed microchip. (d) Removable-top microchip at different upstream pressures. Data shown is from three technical repeats in all the cases.

Figure 3a shows the volumetric flow rate measured in the enclosed and removable-top microchips as a function of the applied pressure. In the case of the enclosed microchip, the flow rate showed a linear and highly reproducible (standard deviation, SD = ± 0.7 μL/min) dependence on the upstream pressure up to 600 kPa. A simple Poiseuille estimation of the flow rate is also shown in Figure 3a, which demonstrates a negligible deviation of the experimental flow rate from the estimation. In comparison, the removable-top microchip showed a linear dependence only up to 200 kPa and then started to leak. In the latter case, the flow rate was also lower than the theoretical estimate. Thus, to ensure a leakproof measurement, the applied pressure was kept below 150 kPa for the removable-top microchips.

We then compared the streaming current magnitude and noise of the devices as a function of applied pressure. Figure 3b shows a comparison of the streaming current for the two freshly cleaned microchips. Since the geometries of the channels are identical in both the designs, we hypothesize that the streaming current magnitude will be a measure of their relative surface qualities. It should be noted that unlike the enclosed microchip, the removable-top version is confined by three active surfaces. The top surface (i.e., PDMS) does not contribute to the streaming current generation as much as the SiO_2_ surfaces due to the inertness of PDMS at physiological pH.^21^ The effect of surface charge on streaming current generation has been well studied.^22^ Therefore, a lower streaming current is expected. As seen, both devices show a linear dependence of streaming current on the applied pressure, underscoring a negligible effect from the electrode polarization or the surface conductivity.^23^

Next, we compared the noise characteristics of the devices. The root-mean-square (RMS) noise was calculated at different streaming currents, i.e., at different upstream pressures. The data are presented as bar plots along with the signal-to-noise ratio (SNR) in Figure 3c,d for the enclosed and the removable-top versions, respectively. The streaming current values at constant upstream pressure are shown in Supporting Information (Section S9). We observed that the RMS noise increases with increasing streaming current. As seen, the RMS noise for the enclosed microchip remains similar (7 pA with standard deviation below ±2 pA, *n* = 3) up to 300 kPa of applied pressure but then sharply increases, reaching 21 pA at 600 kPa. Similar RMS noise behavior was observed in the case of the removable-top microchip. The maximum RMS noise for the removable-top microchip was below 5 pA, which is lower than the enclosed microchip at the same applied pressure. The comparison of SNR for the two designs clearly suggests that the removable-top chip has a higher SNR at all the pressure ranges despite having a lower active surface area. It is important to point out that the comparison of the microchips was done at different pressure ranges considering their different pressure tolerances and SNR values, as presented in Figure 3. The pressure ranges were also chosen to use the optimum Δ*I*_str_. The noise RMS after the surface functionalization at the maxima of the pressure pulses for enclose and removable-top microchips, respectively, were defined as the minimum detectable signal (MDS) for both devices.

### Sensing Performance of the Microchips

**Figure 4:**
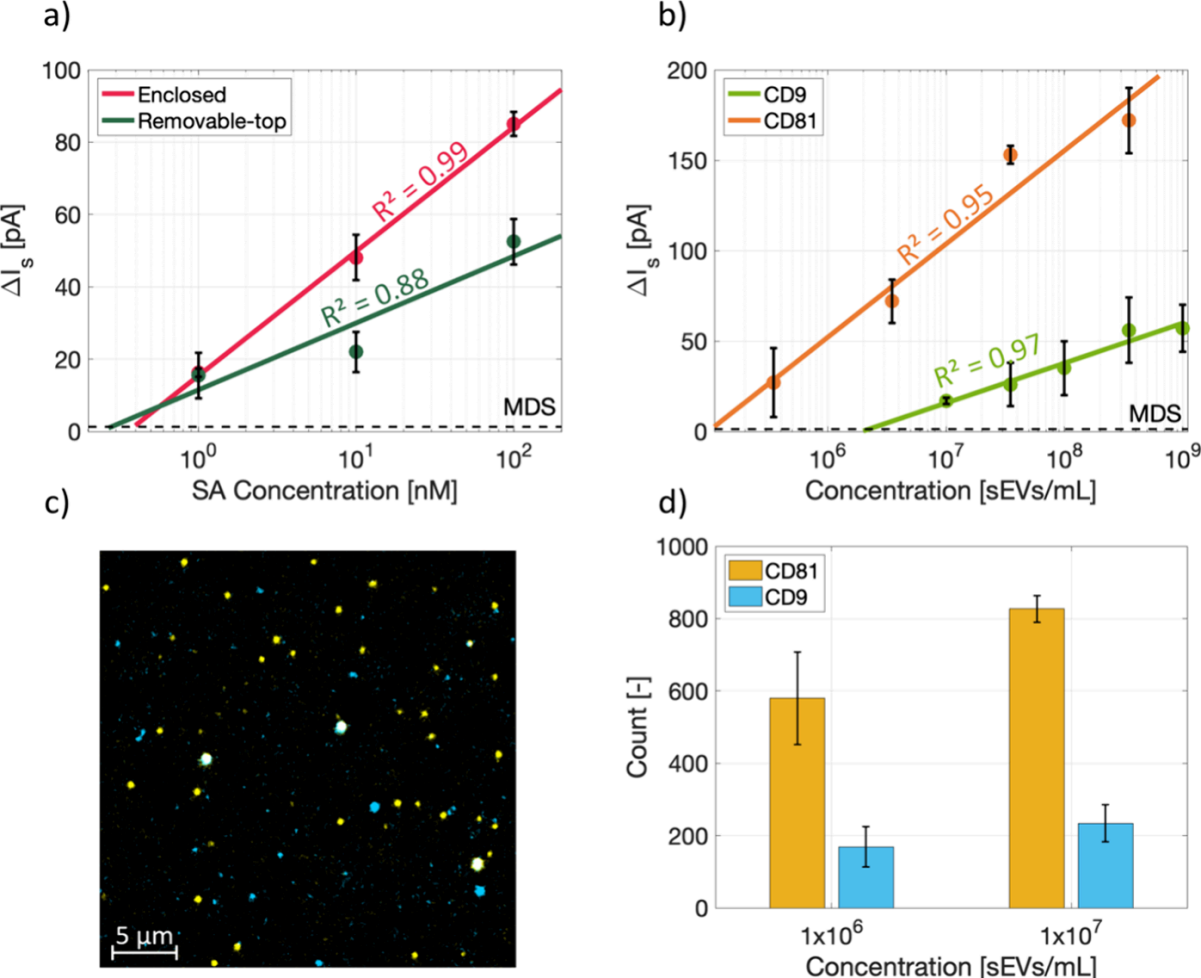
Evaluation of the sensing performances of the microchips. (a) SA concentration measurements using enclosed and removable-top microchips, *n* = 3 technical repeats. (b) Multiplexed detection of sEVs isolated from H1975 cell culture media targeting CD9 and CD81 using the removable-top microchip. The data shown is from one biological replicate, and the error bars represent SD from three technical replicate measurements. (c) A representative single sEV fluorescence microscopy image of the same sEVs as presented in (b), with blue spots indicating sEVs expressing CD9 and yellow spots representing CD81 expressing sEVs. (d) Comparison of sEV counts expressing CD9 and CD81, respectively, when two different concentrations of sEVs/mL were used; error bars indicate the SD for 10 images. In (a) and (b), the horizontal lines represent minimum detectable signal and *R*^2^ shows the goodness of fit.

The sensing performance of the microchips was first investigated by using the biotin–SA pair. To compare the performances, different concentrations of SA were detected using the PPB-based functionalization on both the microchips. Figure 4a shows the response (Δ*I*_s_) of the microchips as a function of the SA concentration. The signal from 1 nM SA was well above the MDS for both devices. The calibration plot intersecting the MDS line shows LoDs of 0.57 and 0.45 nM for the enclosed and removable-top microchip, respectively. The coefficient of determination for linear regression (*R*^2^) reveals how well the fitted line characterized the dynamic range of the microchips in detecting SA. Clearly again, the removable-top configuration shows similar performance as the enclosed version despite their differences in terms of the active surface area. Moreover, the LoD of the enclosed and the removable-top microchips were lower by a factor of 3.7 and 4.9, respectively (see the Supporting Information (Section S10)), compared to the capillary-based detection used in our previous studies.^10,11^

Using the transparent window design, we performed fluorescence-based analysis of the microchip surface (Supporting Information (Section S11)) using Atto 565-conjugated SA. Furthermore, we compared the capture density and uniformity of the different designs. For this purpose, the surface of the devices was coated with PPB and fluorescently tagged SA, under identical conditions. The results presented in Supporting Information (Section S12) shows a nonuniform coverage of the FL-tagged SA along the microchannels in case of the enclosed microchip and a uniform coverage along the removable-top device. As shown in Figure S10, possibly a low surface activation level in the case of the enclosed microchip leads to a nonuniform coverage of the probe density and target molecules along the microchannel as compared to the removable-top microchip. This is likely attributed to the suboptimal cleaning and functionalization of the enclosed microchip compared to the removable-top configuration due to restricted access of the sensing surface in the former case.

With the clear advantage of the removable-top version over the enclosed microchip, we proceeded to utilize the removable-top configuration for sEV analysis. We analyzed the detection sensitivity of sEVs isolated by SEC from the cell culture medium of NSCLC H1975 cells. Prior to the electrokinetic measurements, the isolated sEVs were first characterized using an NTA for their particle/mL concentration, size, and zeta potential (Supporting Information S13). The mean diameter of the particles in the samples as well as their zeta potential were measured to be 153 ± 9 nm (range: 30–200 nm) and −25.83 mV, respectively. The measured size suggests that the isolated EVs to a large extent was sEVs, as defined by MISEV guidelines.^24^Figure 4b shows the calibration plot depicting the signal (Δ*I*_s_) vs sEV concentration. The measurements were performed using the multiplexed configuration of the removable-top microchip simultaneously targeting CD9 and CD81 transmembrane proteins for a large range of sEVs/mL concentrations. The estimated LoDs for CD9 and CD81 detection were 2.2 × 10^6^ and 1.2 × 10^5^ sEVs/mL, respectively. The control measurements are presented in the Supporting Information (Section S14). The observed LoD for CD9 is 2.2 times lower than the value we earlier reported on using the same sensing method on commercial capillaries.^10^ Furthermore, Figure 4b suggests that the number of captured CD81-positive sEVs is higher than the CD9-positive sEVs in the studied sample. Such result could either be due to a higher expression level of CD81 on the studied sEVs or be a consequence of differences in antibody affinity toward their targets. Next, we studied these proteins on sEVs using a fluorescence-based single-EV analysis. A representative fluorescence image depicting single sEVs stained with VioBlue-CD9 and APC-CD81 is shown in Figure 4c. The estimated numbers of CD9- and CD81-positive sEVs in a total of 10 images as a function of concentration are shown in Figure 4d. As seen, the CD81-positive sEVs were more abundant than CD9-positive ones in this sample, thus supporting the observed sensor response.

### Enhancement of LoD

**Figure 5:**
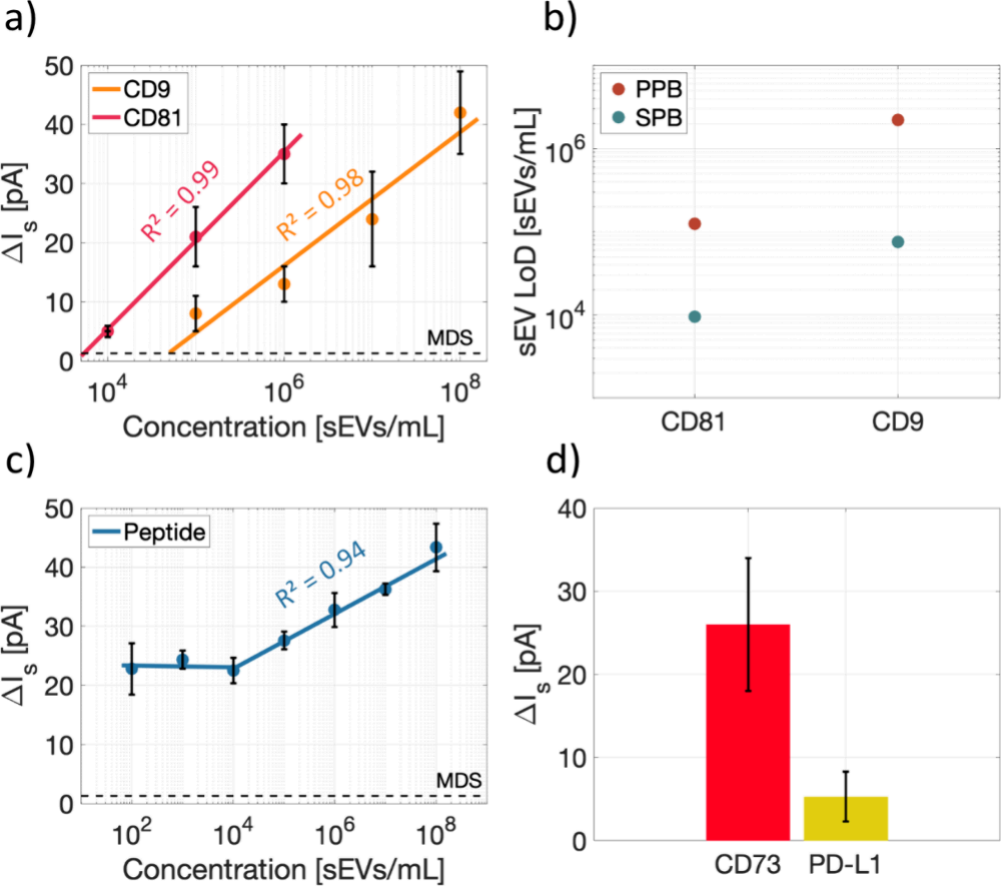
Sensing performance of the removable-top microchip. For (a–c), the sEVs were isolated by SEC from cell culture media of H1975 cells. One biological replicate was used. The horizontal lines are the corresponding minimum detectable signal, and the goodness of the fit is indicated in the plots by *R*^2^. (a) Concentration curve for CD81 and CD9 on the SPB-SA surface, *n* ≥ 3 technical repeats. (b) Limit of detection comparison between PPB and SPB surfaces on the removable-top microchip. (c) Electrokinetic signal for H1975 sEVs captured by membrane-sensing peptides (MSPs) on the SPB surface. The lowest two concentrations are out of the sensing dynamic range of the system, *n* ≥ 3 technical repeats. (d) Multiplexed CD73 and PD-L1 signal from NSCLC patient plasma isolated by SEC prior to treatment. Data shown are from three technical repeats.

To further improve the LoD, we investigated if the choice of the chemical functionalization strategy can improve the sensitivity of the device. We therefore compared two common chemical surface functionalization strategies involving SPB and PPB. First, two removable-top microchips were functionalized by PPB and SPB to compare the noise RMS and SNR. The results are presented in Supporting Information (Section S15). The noise RMS dropped below 2 pA (SD < 0.3 pA, *n* = 3) in both the cases and consequently improved the SNR as compared to the cleaned microchips. The SPB-based functionalization showed a slightly higher SNR as compared to the PPB-based functionalization. Figure 5a shows the signal versus concentration plot of CD9 and CD81 detection of sEVs for the removable-top microchip using an SPB-based surface functionalization. An identical method and sample as used in Figure 4b was applied here. As seen, the LoD of the removable-top chip was significantly lower compared to that of the enclosed microchip. For better comparison, the LoD obtained with the two functionalization methods is presented in Figure 5b. As seen, the SPB-based method allows to reach an LoD of 9.5 × 10^3^ sEVs/mL in case of CD81 and 7.6 × 10^4^ sEVs/mL for CD9, both of which are lower by factors of 13 and 29, respectively, as compared to PPB-based functionalization and a factor of 65 times lower than the capillary-based method, which we used before for detection of CD9 on sEVs from the same cell line.^10^ Furthermore, the optimized surface functionalization resulted in a very low sensor response for the negative controls, as shown in Supporting Information (Section S14).

Although the analysis of sEV membrane protein expression has high clinical importance, immunocapture may be influenced by the heterogeneity in sEV surface expression, e.g., tetraspanins,^25,26^ and hence the relative abundance of a certain sEV population could be influenced by using immunocapture with antibodies. Besides, the affinity of the selected antibody toward its target may differ depending on their origin. Therefore, we further studied the detection sensitivity of the device using membrane-sensing particles (MSPs) for sEVs capturing.

MSPs directly bind to the sEV membrane regardless of a certain protein expression pattern and thus provide a more accurate estimation of the sEV concentration. Thus, the MSPs selected and applied here have previously been reported^13^ to capture sEVs in the size range of 50–130 nm irrespective of their surface protein profiles as they have affinity for a highly curved lipid membrane, which can be considered to be a shared “epitope” for nanovesicles. Figure 5c shows the calibration curve obtained with the removable-top-chip and SPB-based functionalization. As seen, an LoD of 10^4^ sEVs/mL was obtained in that case before the applied sEV concentration exited the dynamic range of the sensor.

To demonstrate the prospect of clinical applications, we analyzed programmed death-ligand 1 (*PD-L1*) and cluster of differentiation 73 (CD73) also known as ecto-5′-nucleotidase (NT5E) expression on sEVs isolated by SEC from plasma of an NSCLC patient with advanced disease (for details, see Materials and Methods section). The NTA assessment of the particle size revealed a mean size of 101 ± 0.6 nm, which is within the sEV size range.

PD-L1 was chosen for these analyses since immune checkpoint inhibitors (ICI) targeting the PD-L1/PD-1 association are used for treatment of a subset of NSCLC patients and given that it has been reported that PD-L1 expression on EVs (both small- and large-sized EVs) isolated from plasma or serum of NSCLC patients may hold prognostic potential.^27,28^ We also examined CD73, which is well known to cause an immune suppressed tumor microenvironment and which recently was demonstrated to take part in adenosine generation when expressed on EVs/exosomes isolated from cancer patient serum, thereby also impacting on ICI treatment response.^29−32^

On the microchip, an SPB surface functionalization was employed to benefit from the lower limit of detection. The streaming current signal in detecting CD73 and PD-L1 for this sample at 1 × 10^7^ sEVs/mL (as determined by NTA) is shown in Figure 5d. The negative control showed a signal lower than the MDS of the microchip, similar to the cell line sEV measurements in the previous section. Of note, both PD-L1 and CD73 expression on sEVs extracted from the NSCLC patient plasma sample was also confirmed by analyzing their expression by PEA on a total sEV protein extract from a replica isolation of the same plasma sample as used in Figure 5d. Thus, with respect to PD-L1 the sEVs from NSCLC patient plasma had a linearized NPX value of 24.0 while the RIPA negative control was 1.2, confirming PD-L1 expression in the studied sEVs. Similarly, CD73 gave a clear signal from the sEVs sample above the RIPA negative control with linearized NPX values of 2578.3 and 1.4, respectively.

## Discussion

The detection method applied here has been widely studied^11,19,33^ and improved by many research groups including our team. In addition, we have earlier explored the diagnostic opportunities of the method,^11^ albeit in a laboratory setting. Translation of these technical developments for potential use in a clinical diagnostic setting with a viable microchip is the obvious next step. We present here such a microchip that can be mass produced on silicon wafers. We used different characterization methods and bioanalysis, to address three main aspects: (i) the design aspect and its influence on the device performance, (ii) the impact of the chemical functionalization strategies on the device characteristics, and (iii) the sensitivity and the LoD of the sensors in comparison to other methods. It should be noted that the streaming current measurements are highly sensitive to various parameters such as the size and charge of the target, the pH of the measurement buffer,^12^ and the choice of the functionalization technique.^10^ Hence, it is important to optimize the experimental conditions, such as the choice of surface functionalization and antibody, before any measurement.

### Design Aspect and Its Influence on the Device Performance

The advantage of silicon-based technologies for scalable fabrication is well established.^34^ Besides, the SiO_2_ surface has also been widely studied for the immobilization of affinity probes, thus justifying the selection of material and process technologies reported here. As presented in the Supporting Information (Section S3), streaming current increases proportionally with the pressure difference. Hence, an obvious design choice would be a mechanically robust microchannel like the enclosed microchip presented in this work, which can withstand a higher pressure. As seen in Figure 3b, while Δ*I*_str_ expectedly increases with Δ*P*, it does not necessarily translate to increasing SNR (Figure 3c) for the entire range of Δ*P*. Since the noise RMS is roughly constant at low pressures and streaming current scales with the upstream pressure (Figure S7), the SNR increases at the beginning and then appears to reach a plateau before dropping at a higher Δ*P*. While the mechanism behind such a noise behavior requires further analyses, which is beyond the scope for the present work, it motivated us to construct and examine a removable-top version of the microchip that only operates in the low Δ*P* region (Figure 3a,b). However, unlike the enclosed design of the microchip, the removable-top configuration allows full access to the sensing surface, making it more convenient and compatible with different surface functionalization strategies including automated printing of the affinity probes.^35^ As seen in Figure S7 and Figure 3d, the removable-top configuration also produced a similar *I*_str_ at a Δ*P* of 150 kPa but higher SNR due to lower noise RMS. Given that the removable-top design has 35% less active surface than the enclosed version, the obtained result is interesting and may suggest that this microchip configuration likely has a higher surface activation level than the enclosed version. Besides, the LoD comparison presented in Figure 4a clearly suggests that the removable-top design is better suited for sensing applications.

### Impact of the Chemical Functionalization Strategies on the Device Characteristics

**Figure 6:**
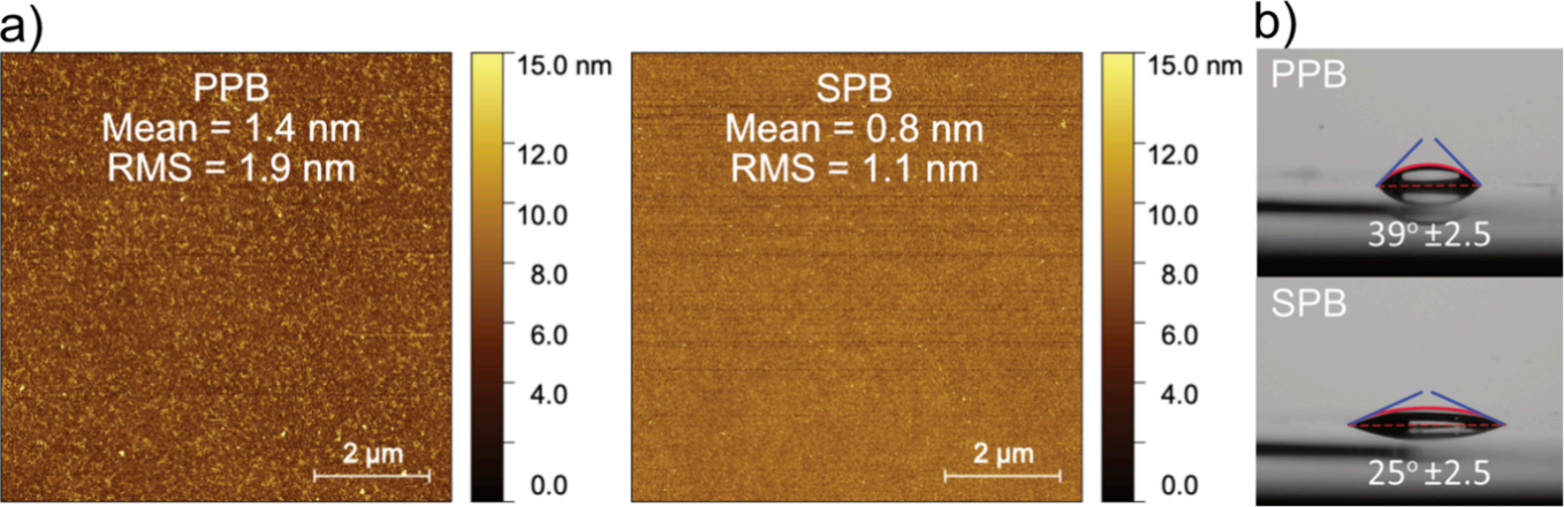
Comparison of the surface roughness and hydrophobicity of the PPB and SPB surfaces. (a) AFM images of SPB and PPB surfaces comparing the mean and RMS of the roughness. The higher roughness of the PPB surface could be a possible reason for its failure to detect very low concentrations of analytes. (b) Contact angle comparison between PPB and SPB surfaces, demonstrating that the PPB surface is more hydrophobic.

Streaming current-based approaches also critically depend on the choice of linker molecules that bind the affinity probes to the surface. This is primarily due to the influence of surface roughness and charge contrast between the analytes and the surface, as we earlier reported.^10^ To further improve the performance of the device, we investigated the relative influence of the PPB- vs SPB-coated surface. The SPB-based strategy allowed us to lower the LoD by more than a factor of 10 over the PPB-coated surface (Figure 5b). To investigate this further, we performed AFM and contact angle measurements, as presented in Figure 6. For this, we used two silica coverslips, which were functionalized by PPB and SPB. The mean roughness for the SPB- and PPB-coated surface was 0.8 and 1.4 nm, respectively (Figure 6a). It is known that the surface roughness in the order of the Debye length can reduce the influence of particle adsorption on the generated streaming current.^22^ This may explain the observed higher LoD for the PPB-coated surface. Furthermore, the contact angle measurement shows a 14° difference between PPB and SPB surfaces, indicating that the PPB surface was more hydrophobic (Figure 6b). This means that the ions in the electric double layer likely will slip faster on the surface and generate a higher absolute value of the streaming current.^36,37^ Slide angle measurements comparing the friction force between the liquid and the surface were also carried out, Supporting Information (Section S16), and supported this claim. A higher streaming current generation on the PPB surface could also lead to a steeper slope in the concentration curves by increasing the sEV concentration. This is evident when comparing the same markers of the sEVs on both the PPB and SPB surfaces.

### Sensitivity and LoD of the Sensors in Comparison to Other Methods

The data presented in Figures 4 and 5 clearly suggest that the fabricated microchip offers a far better LoD compared to what we previously reported using the same principle but with commercial capillaries.^10^ A key advantage of the method stems from its dependence on the size of the analytes, an aspect which we have previously investigated.^12^ Thus, analytes such as sEVs are suitable candidates to be monitored by the method as it offers an extremely low detection limit. A major challenge is, however, the heterogeneity of sEV samples with respect to their membrane protein expression levels and composition. Therefore, to ensure similar conditions, the comparison with our previous studies had to be done using only identical samples and antibodies. This is, however, difficult to maintain while comparing among different methods reported by different groups. In this context, MSPs may be a more suitable alternative as they are able to enrich small vesicles on the basis of specific membrane biophysical traits, opposed to the preselection of sEV subpopulations introduced by the use of antibodies.^13,15^ As presented in Figure 5c, an LoD of 10^4^ sEVs/mL could be achieved using such peptide-based capture and monitoring of sEVs isolated by SEC from cell culture media of the NSCLC cell line H1975. Although a direct comparison of LoD among different devices is difficult, a qualitative assessment may still be possible. Table 1 shows the state-of-the-art techniques and their reported LoD for EVs, sEVs, or exosomes compared to the present work. Clearly, the proposed method is among the best performing methods.

**Table 1:** Limit of Detection and Linear Range Reported in the Literature Targeting EVs or Exosomes on Different Platforms

platform	LoD [particles/mL]	linear range [decades]	ref
this work	1 × 10^4^	4	N/A
iMEX	3 × 10^4^	4	(38)
covalent organic framework	1.6 × 10^5^	5	(39)
colorimetry	2.76 × 10^6^	1	(40)
colorimetry	5.2 × 10^8^	1	(41)
electrochemiluminescence	7.41 × 10^4^	3	(42)
electrochemistry	2.09 × 10^4^	7	(43)
electrochemistry	2 × 10^5^	4	(44)

## Conclusions

In conclusion, we demonstrated the fabrication and characterization of a novel microchip-based electrokinetic biosensor. The devices were fabricated on a silicon platform using a scalable process technology. We investigated different aspects of the microchip including the design considerations as well as electrical and fluidic behavior and their relative performance with respect to biosensing, thus giving a practical and necessary guideline to further develop and implement such a biosensor. A custom-built chip manifold was also constructed for an easy interfacing of standard fluidic connectors with the microchips and for a leak-free flow of electrolytes. The sensitivity and LoD of the microchips were compared with previous reports demonstrating their superior performance. Particularly, for the detection of sEVs, we show that the developed microchip allows for a more than 60× lower LoD than the previous reports using the same principle. Moreover, our analysis of sEVs isolated from plasma of an NSCLC patient and where two targets of relevance for treatment were explored, PD-L1 and CD73 respectively, illustrates the future prospect of the microchip in a clinical setting of oncology.

## Supporting Information Available

Extracellular vesicles from cell culture media—isolation and characterization; non-small cell lung cancer plasma sample—isolation, characterization, and proximity extension assay profiling of extracellular vesicles for PD-L1 and CD73; theoretical background; experimental setup; twin reservoir and agitating platform; effect of cleaning; workflow of the fabrication process; white light interferometry characterization of the microchip surface; streaming current values at constant upstream pressure; detection of streptavidin by different devices; fluorescent streptavidin measurement on an enclosed microchip; non-uniform coverage of the FL-tagged SA on the enclosed microchip; characterization of particle size of the extracellular vesicles from non-small cell lung cancer cell culture media by nanoparticle tracking analyses; control measurements on the PPB and SPB devices; and noise RMS and SNR comparison of the clean, PPB, and SPB functionalization strategies; slide angle measurement (PDF)
