# Supplementary material for: Design and Optimization of a Silicon-Based Electrokinetic Microchip for Sensitive Detection of Small Extracellular Vesicles
Source: ACS Sens. 2024 Jun 7;9(6):2935–45. doi: 10.1021/acssensors.4c00110 (PMC11217933; doi:10.1021/acssensors.4c00110)
Supplement: Supplementary file 1 — se4c00110_si_001.pdf [file se4c00110_si_001.pdf]

Supplementary information: Design and optimization of silicon-based electrokinetic microchip for sensitive detection of small extracellular vesicles

Moein Talebian Gevari [1,*], Siddharth Sourabh Sahu [2], Fredrik Stridfeldt [2], Petra Hååg [3], Luigi De Petris [3,4], Kristina Viktorsson [3], Rolf Lewensohn [3,4], Alessandro Gori [5], Marina Cretich [5], and Apurba Dev [1,2,*]

[1] Division of Solid-State Electronics, Department of Electrical Engineering, Uppsala University, 75121 Uppsala, Sweden.

[2] Department of Applied Physics, School of Engineering Sciences, KTH Royal Institute of Technology, 10691 Stockholm, Sweden.

[3] Department of Oncology-Pathology, Karolinska Institutet, S-171 64, Solna, Sweden.

[4] Theme Cancer, Medical Unit head and neck, lung, and skin tumors, Thoracic Oncology Center, Karolinska University Hospital, SE-171 64, Solna, Sweden.

[5] Consiglio Nazionale delle Ricerche, Istituto di Scienze e Tecnologie Chimiche "Giulio Natta" (SCITEC), Milan, Italy.

* Corresponding author

E-mail address: moein.talebian@angstrom.uu.se (M.T. Gevari), apurba.dev@angstrom.uu.se (A. Dev)

S1. Extracellular vesicles from cell culture media- Isolation and characterization

The sEVs used in this study was harvested from cell culture media of the non-small cell lung cancer (NSCLC) cell line H1975 (ATCC® CRL-5908™, distributor LGC Standards, Middlesex, UK) essentially as previously been described.[1]

Thus, sEVs were obtained from RPMI 1640 medium where cells were grown in the presence of fetal bovine serum (FBS) but where endogenous exosomes have been depleted. The sEVs were isolated using size-exclusion chromatography (SEC) on qEVoriginal Gen 2, 70nm columns (Izon Science, Oxford, UK) in the same manner as described in Stiller et al.[1] The particle size and zeta potential were characterized by nanoparticle tracking analysis (NTA, Zetaview from particle Metrix). For these analyses the sEVs samples were diluted 1:100 in filtered PBS. The sample was injected in 100 µL portions for three times and the cell temperature was maintained at 24° C. The size analysis was done on eleven positions in the sample cell while for the zeta potential a two-position measurement was performed. The CD9 expression on the sEVs used in this work were not studied by western blot specifically but CD9 has been confirmed to be expressed in sEVs from H1975 cell culture media when the SEC isolation was used.[2]

S2. Non-small cell lung cancer plasma sample- isolation, characterization and proximity extension assay profiling of extracellular vesicles for PD-L1 and CD73

For the sEVs isolation, 0.35 mL of frozen plasma was used and first centrifuged at 720 rcf for 10 min (Mikro 200R, Hettich, Stockholm, Sweden) followed by filtering through a 0.22 µm syringe filter (Acrodisc®, 13 mm diameter, Pall Corporation, VWR, Spånga Sweden). The sample was diluted in PBS to 0.5 ml and loaded onto a size exclusion chromatography column (see S1). For this experiments fraction 1-5 (obtained by an Automatic Fraction Collector (AFC2; Izon Science)) each containing 0.5 mL were pooled and concentrated to ~0.12 mL. Particle size and concentration in the sample was assessed by nanoparticle tracking analysis (NTA) using the NS300 equipment (Malvern Panalytical, Malvern, United Kingdom). For the evaluation, 5 videos of 60 s were used with the following settings: camera level 12, detection threshold 5, average particles/ frame 24. The mean size of the particles was 101.0 +/- 0.6 nm and the mode size were 80.0 +/- 1.8 nm. The concentration was $4x10^{10}$ sEVs/ml. For the microchip assessment this solution with particles in 1xPBS was diluted to $1x10^{7}$ sEVs/mL.

The PD-L1 and CD73 assessment in sEVs carried out using proximity extension assay (PEA) (Olink Proteomics AB, Uppsala, Sweden) according to the manufacturer's instructions by the Affinity Proteomics Uppsala facility, SciLifeLab, Uppsala University, Sweden. For the PEA analyses of the plasma-isolated sEVs, the sEVs which were kept in PBS, were lysed in 10x RIPA buffer to give 1x RIPA (50 mM Tris-HCl, pH 7.4, 150 mM NaCl, 1% Triton X-100, 5 mM EDTA pH 8, 0.1% SDS) and the total sEVs protein cargo was analyzed. The concentration of particle/EVs as assessed by NTA (data not shown) after dissolving the sample in RIPA buffer was $1.9x10^{7}$ particles/µL and from this the PEA assay were conducted per manufacturer's instructions. A negative control, 1xRIPA buffer without any sEVs was applied. For the PD-L1 protein expression, data was taken from the Immune Oncology® panel while for the CD73 expression (Olink name: 5'-NT), the data was obtained from Oncology II® (for full details of markers see manufacturer homepage). After analyses with the Olink Wizard for GENEX software (MultiD Analyses AB, Gothenburg, Sweden), the resulting Normalized Protein eXpression (NPX) values were used for the evaluation of results which are presented as linearized values for PD-L1 or CD73 respectively in the result section.

S3. Theoretical background

The potential difference of the ions and electrons on the solid/electrolyte interface creates the electric double layer (EDL). A pressure gradient between the two ends of the microchannel pushes the electrolyte along the charged surface inducing streaming current (I_s) that can be defined as the charge flowing through the interface (S) per unit of time (Eq. (1)).[3]

$$I_s = \iint_S \rho_e V . dS \tag{1}$$

where ρ_e and V are electric charge density and fluid velocity, respectively. Governing the charge density by Poisson-Boltzmann equation and the laminar flow in the microchannels by Stokes equations, one could express streaming current as in eq. (2):[3]

$$I_s = \varepsilon \varepsilon_0 \frac{A}{\eta} \frac{P}{L} \zeta \tag{2}$$

where $\epsilon\epsilon_0$ and η refer to the permittivity of the electrolyte and dynamic viscosity, respectively, and L, P, and A refer to the length, upstream pressure, and cross-sectional area of the microchannel, respectively. Finally, ζ corresponds to the interface potential known as zeta potential which is the property of the surface.

As in eq. (1) and (2), the streaming current flowing inside a microchannel depends on the upstream pressure and the surface area contributing to the streaming current generation. In addition, a uniform and dense charge distribution on the interface could increase ζ and affect the induced streaming current.

S4. Experimental setup

A schematic of the experimental setup consisted of a high-pressure pure nitrogen gas capsule regulated by an Elveflow OB1 flow controller coupled with a thermal time of flight (TOF) flow sensor (Elveflow, MSF3) to measure the flowrate during the experiments. The electrolytes were hydraulically pushed by the nitrogen gas into the system through PEEK tubing and microfluidic connections that were obtained from Darwin Microfluidics (Paris, France). A continuous train of pressure pulses with a duration of 30 s, was used to perform a two-point streaming current measurement. The resulting streaming current pulses (ΔI_{str}) were measured by a Keithley picoammeter (model no. 2636A) as a function of time, thus, constituting a baseline measurement while the pressure pulses (ΔP) were applied directly by the pressure regulator.

The measurements in this study involved recording the initial streaming current baseline after the immobilization of the capturing probes (ΔI_{str1}) and the final baseline after the injection of the target (ΔI_{str2}). The signal reported in this work is the difference between the two baselines denoted as ΔI_s (=ΔI_{str2} - ΔI_{str1}). The incubation of sEVs on the microchip was done in 1x PBS to resemble physiological conditions, whereas both the baselines were measured in 0.1x PBS to reduce the charge screening. Custom made Labview and MATLAB scripts were used to record and analyze the data, respectively. The noise-sensitive components of the experimental setup were placed in a Faraday cage. Suitable holes were drilled in the chip manifold with the same diameter as the inlet/outlet ports on the microchips. To eliminate the leakage from the system, silicone rubber O-rings, purchased from Apple Rubber Inc. (Lancaster, NY, USA), with 1 mm inner diameter were placed between the microchip and the manifold.

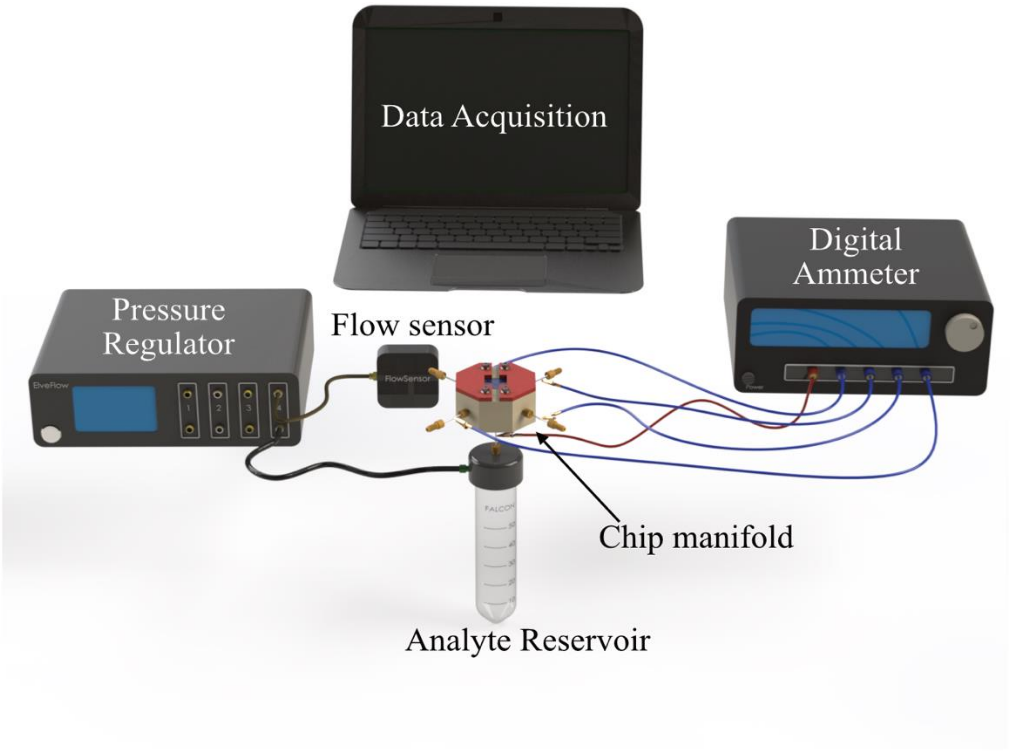

Figure *S1 – The schematic of the experimental setup consisting of the chip manifold, Platinum electrodes, digital ammeter, pressure regulator, flow sensor, analyte reservoir, and data acquisition system.*

S5. Twin reservoir and agitating platform

A PDMS twin reservoir was casted on an in-house designed PEEK mold (see Figure S2-a). Then, it was inserted on the removable-top microchip to isolate two microchannels from the other

two and thereby facilitating the multiplexed measurements (see Figure S2-b). Finally, the PDMS twin reservoir was sandwiched on the microchip to avoid cross contamination across the isolated microchannels. Different solutions were incubated in the two reservoirs while the whole platform was agitating on a benchtop vortexer (see figure S2-c).

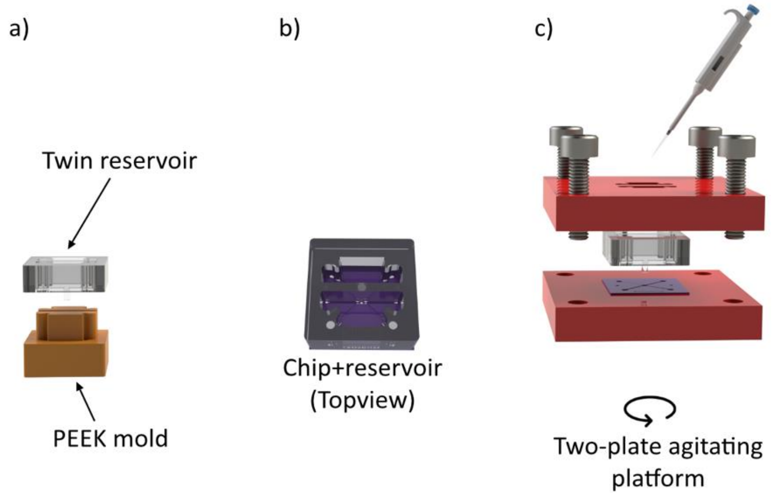

Figure S2 – a) The PDMS twin reservoir casting and b) insertion on the chip along with c) the agitating platform.

To demonstrate the performance of the twin reservoir, a microchip was functionalized by PPB following the protocol stated in the manuscript. Thereafter, one side of microchip was functionalized by FL-tagged SA using the PDMS twin reservoir and the agitating platform. The FL-microscope images in Figure S3 shows a clear distinction between the PPB-SA and PPB covered microchannels. The microchannel with no SA shows no fluorescent signal while the microchannel coated by SA emits a significant fluorescent signal. This demonstrates the successful application of the PDMS twin reservoir to isolate the microchannels and prevent cross-contamination to perform multiplexed detection.

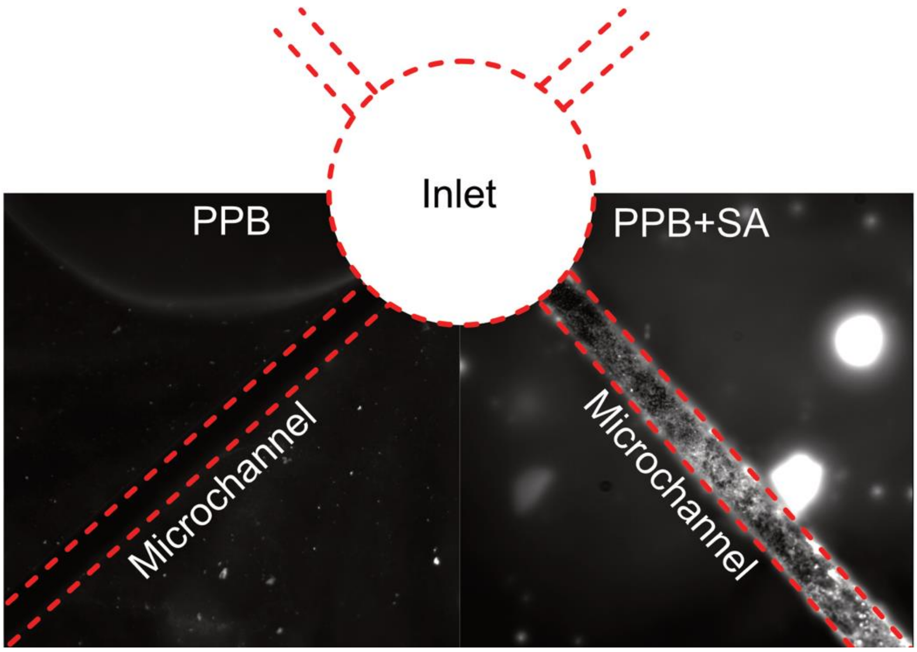

Figure S3 – the performance of the twin reservoir. A PPB covered microchannel beside a SA covered microchannel. The dashed lines show the borders of the microchannels and the inlet port.

S6. Effect of cleaning

RCA1 is a well-established method to remove organic contamination from a SiO_2 surface,[4] thus, activating the surface for hydrolysis reaction.

$$SiOH \overset{pK_a}{\longleftrightarrow} SiO^- + H^+$$

Where pK_a is the acid dissociation constant.[5] Such reactions lead to the generation of surface charges which is necessary for generating streaming current. To demonstrate experimentally, a bare enclosed microchip was used to measure the baseline before and after cleaning by the RCA1 protocol. Figure S4 shows the I_{str} for a pressure pulse between 150 kPa and 300 kPa.

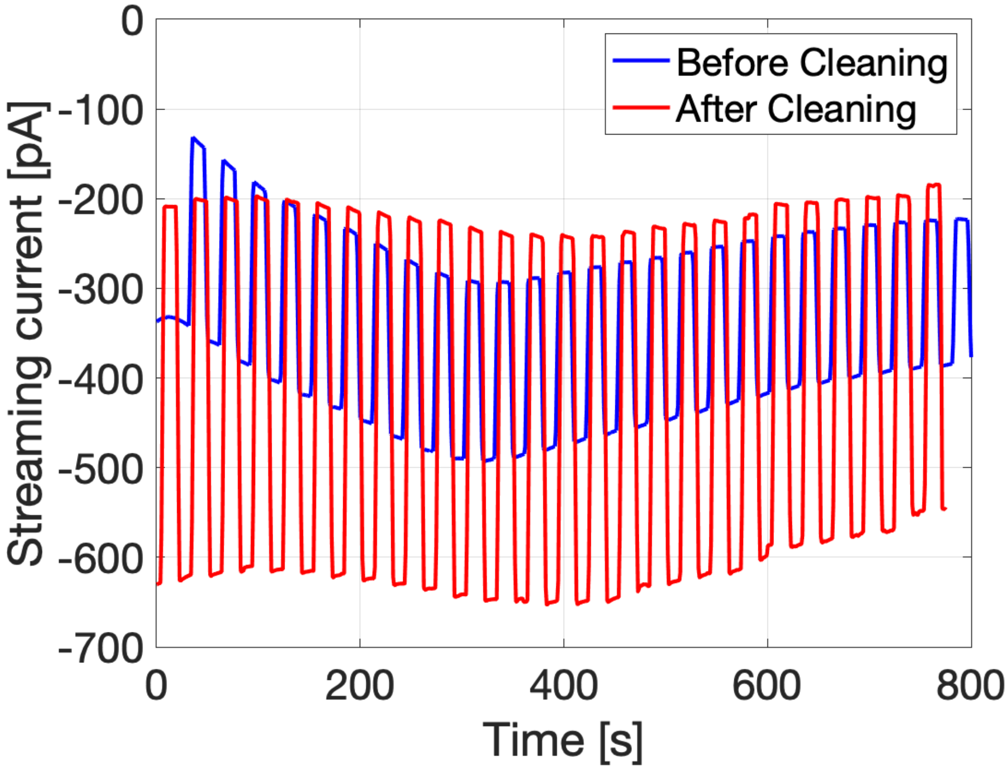

Figure S4 – Recorded streaming current before and after cleaning.

S7. Workflow of the fabrication process

A detailed workflow of the fabrication process is shown in Figure S5.

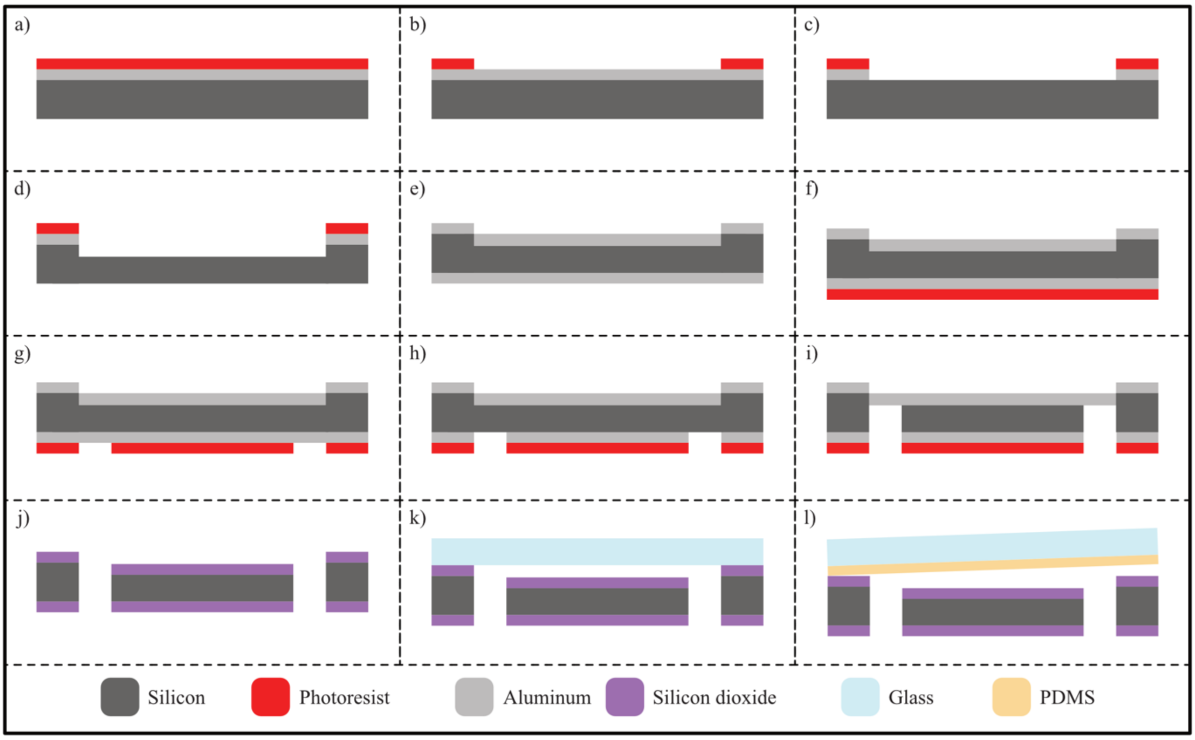

Figure S5 – Cross sectional view of the fabrication process flow, a) silicon substrate covered by aluminum and photoresist, b) the developed photoresists after the first lithography, c) dry etched aluminum layer and open silicon surface, d) dry etched silicon forming the structure of the microchip, e) redeposition of aluminum on both front and back sides, f) spun coated photoresist for the second lithography on the back side, g) developed photoresist after the second lithography, h) dry etched aluminum mask, i) dry etched silicon forming the inlet and outlet ports, j) thermally grown silicon dioxide acting as the active surface of the sensor, k) anodically bonded glass ca, l) PDMS covered glass on the removable microchip.

S8. White Light Interferometry characterization of the microchip surface

To characterize the surface roughness of the microchips by white light interferometry, a removable-top microchip was cleaned using RCA 1 cleaning solution and the structure was characterized by a ZYGO optical profiler as shown in Figure S6. The inset of this figure depicts representative individual roughness elements analyzed on the surface. A total number of about 340K elements were analyzed and the centerline average was 9 nm.

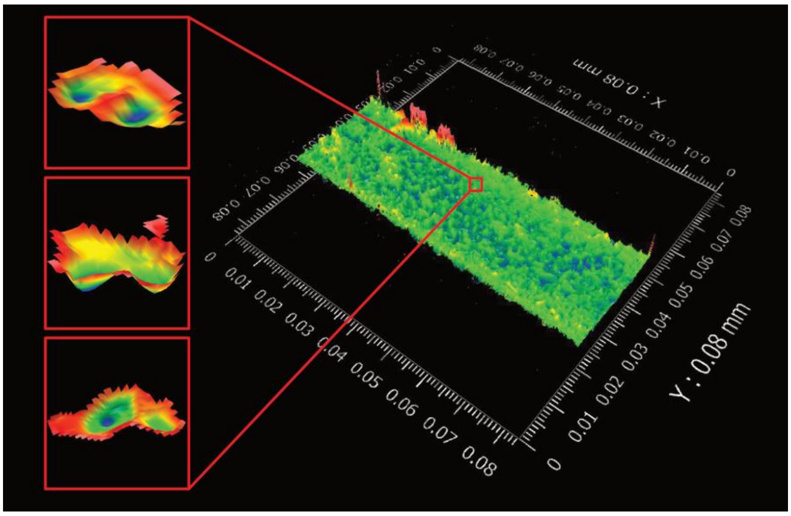

Figure *S6 – White Light Interferometry image of the microchip and the individual roughness elements.*

S9. Streaming current values at constant upstream pressure

To find the noise RMS, the upstream pressure was kept constant and the streaming current was measured. Figure S7 shows the streaming current values.

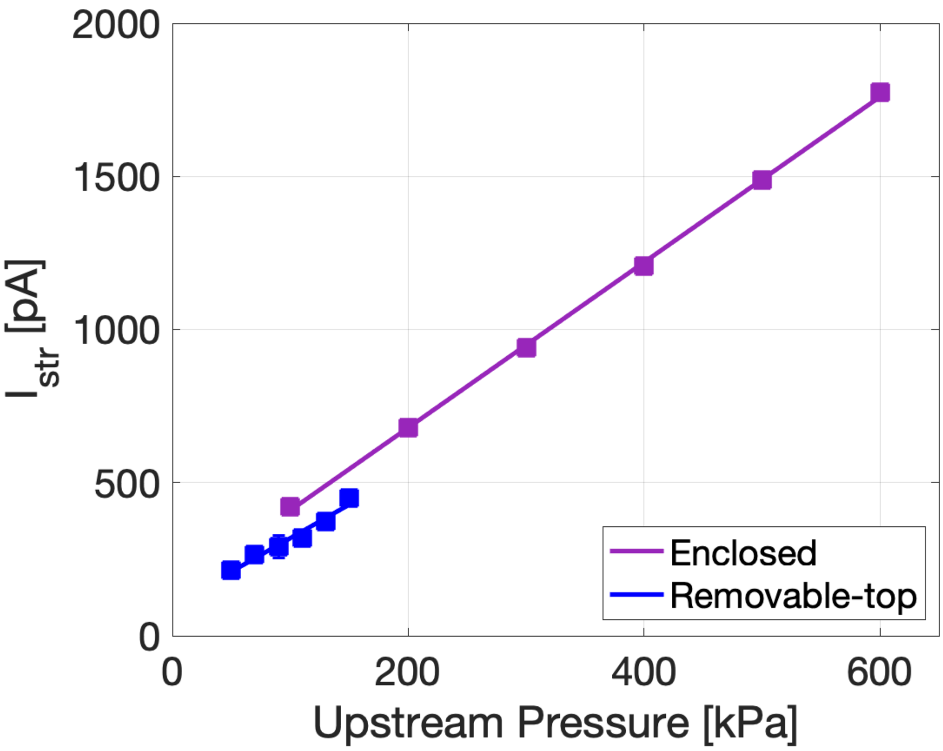

Figure *S7 – streaming current at baseline of the enclosed and the removable-top microchips at different upstream pressures. Data shown is from 3 technical repeats.*

S10. Detection of streptavidin by different devices

The performances of both the enclosed and the removable-top microchips were compared with the commercial silica capillary tubes. The concentration curve in Figure S8 shows a significant LoD enhancement for both microchips as compared to the silica capillaries.

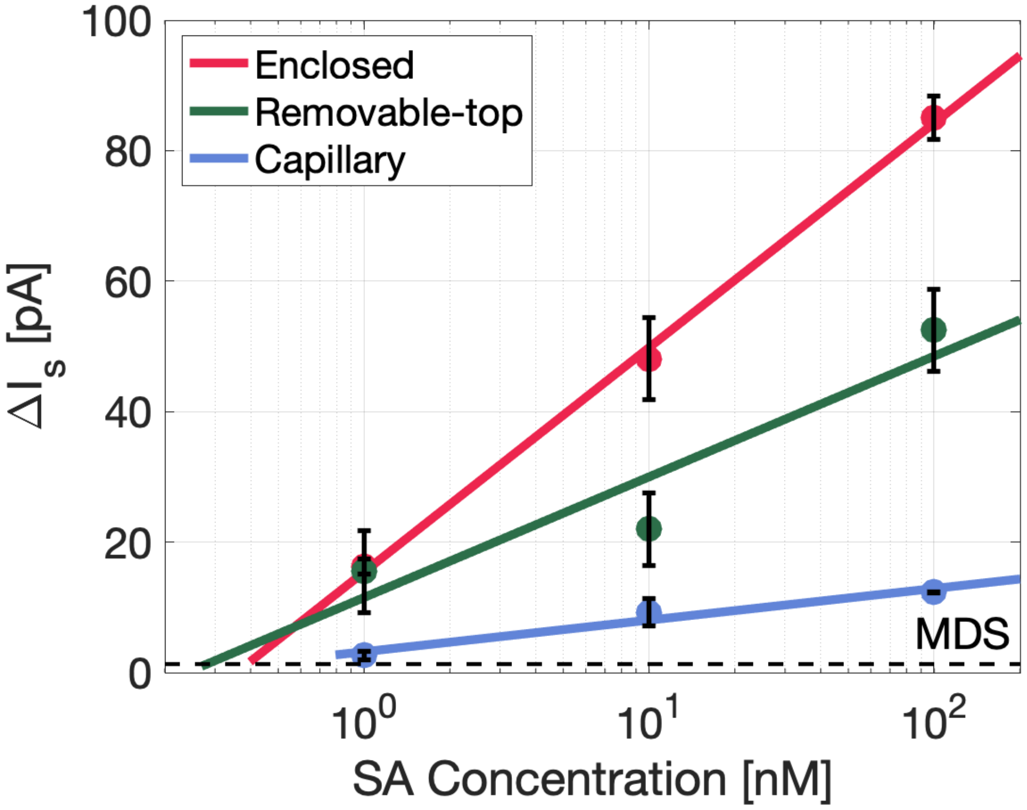

Figure S8 – Concentration curve for SA detection on PPB surface using the enclose microchip, the removable-top microchip, and the commercial capillary tubes. Data shown is from 3 technical repeats in all the cases.

S11. Fluorescent streptavidin measurement on an enclosed microchip

The simultaneous electrokinetic and fluorescent measurements on the enclosed microchip through the optical window in detecting FL-tagged SA is shown in Figure S9.

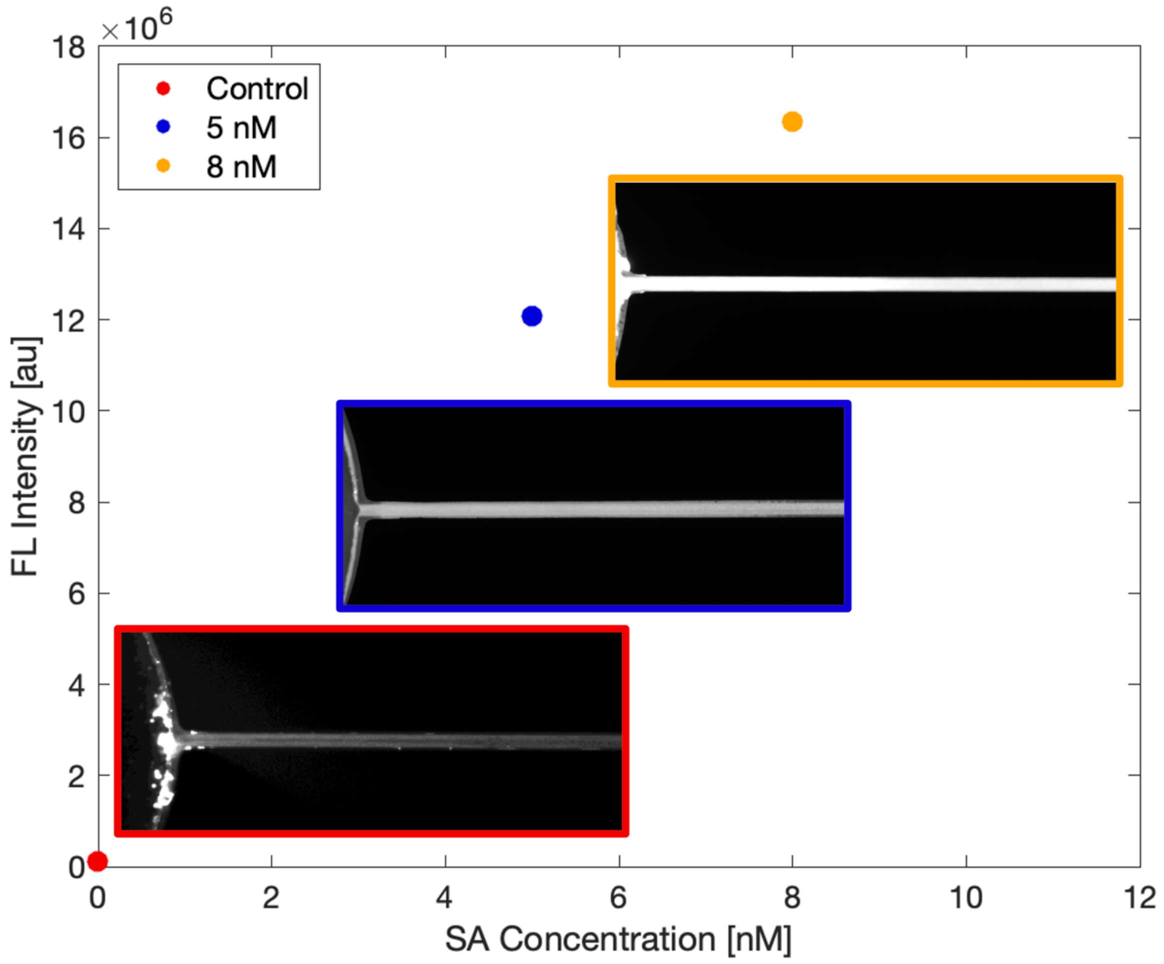

Figure *S9 – Fluorescent intensity of SA on the enclosed microchip.*

S12. Non-uniform coverage of the FL-tagged SA on the enclosed microchip

Due to suboptimal cleaning of the enclosed microchip, the surface is not uniformly activated by the RCA1 cleaning. Therefore, affinity probes do not uniformly cover the microchannels. Figure S10 shows the non-uniform coverage of FL-tagged SA inside the microchannels and the uniform coverage in case of the removable-top microchip. The fluorescent intensity drops significantly the further it gets from the fluidic ports in the enclosed microchip where the cleaning solution could diffuse and activate the surface prior to the measurements. The bottom inset of the figure shows the uniform FL intensity from the immobilized SA on the removable-top microchip.

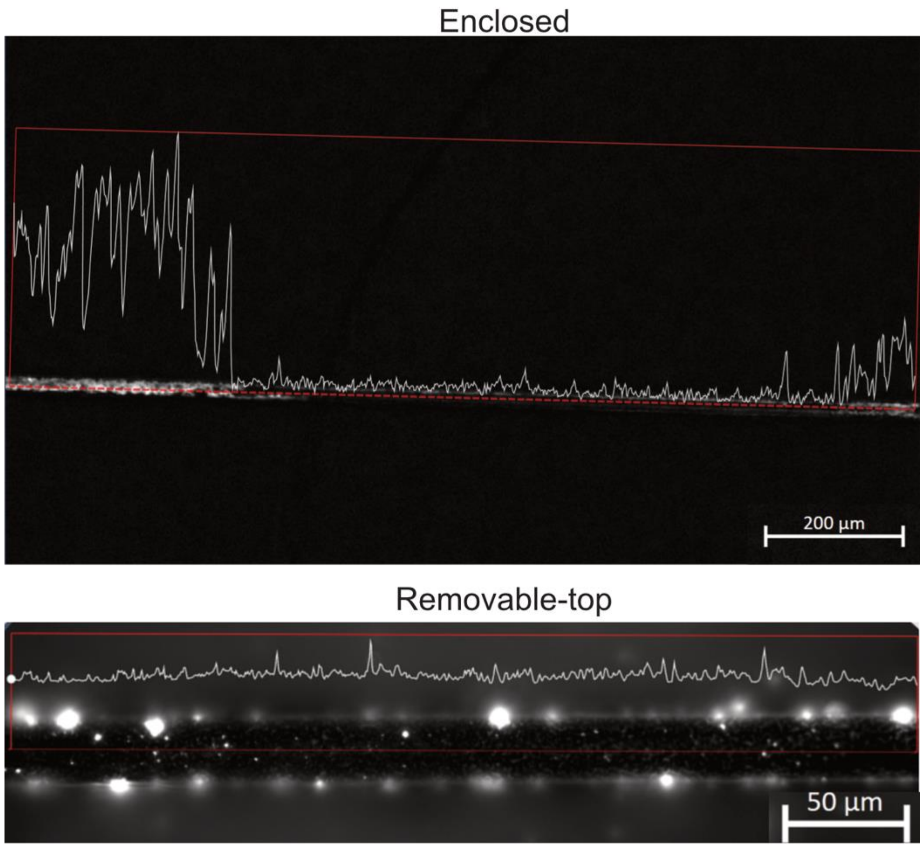

Figure S10 –coverage of the microchannel by FL tagged SA in the enclosed microchip (top) and removable-top (bottom).

S13. Characterization of particle size of the extracellular vesicles from non-small cell lung cancer cell culture media by Nanoparticle Tracking Analyses

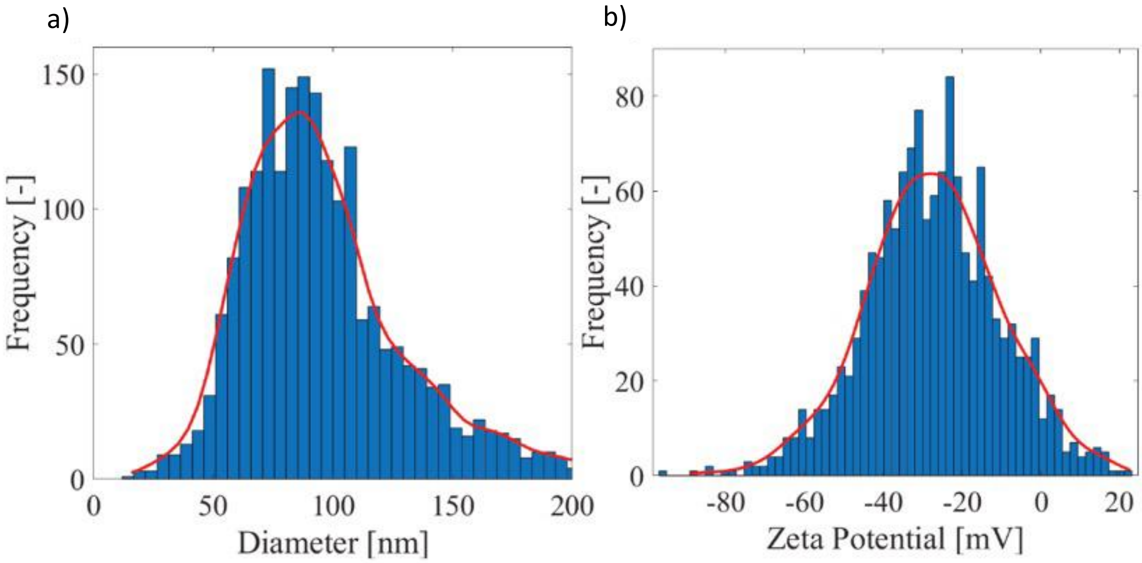

Figure *S11 – a) Particle size distribution (in nm) in the sample of extracellular vesicles (EVs) isolated from cell culture media of NSCLC H1975 cells, b) Zeta potential (in mV) of the sample shown in a.*

S14. Control measurements on the PPB and the SPB devices

For the control measurements, a removable-top microchip was functionalized up to the antibody immobilization step for both PPB and SPB devices. Then 0.01 w% Pluronic-F108 was incubated on the surfaces for 30 minutes to passivate the uncovered surfaces. After washing the first baseline was measured. Then the highest concentration of sEVs (1x10^8 sEVs/mL) was incubated on the microchips and the second baseline was measured. As seen in Figure S12, the control signal on both surfaces is close to the MDS (5 pA). The control signal was subtracted from the sEVs sensing data presented in the manuscript.

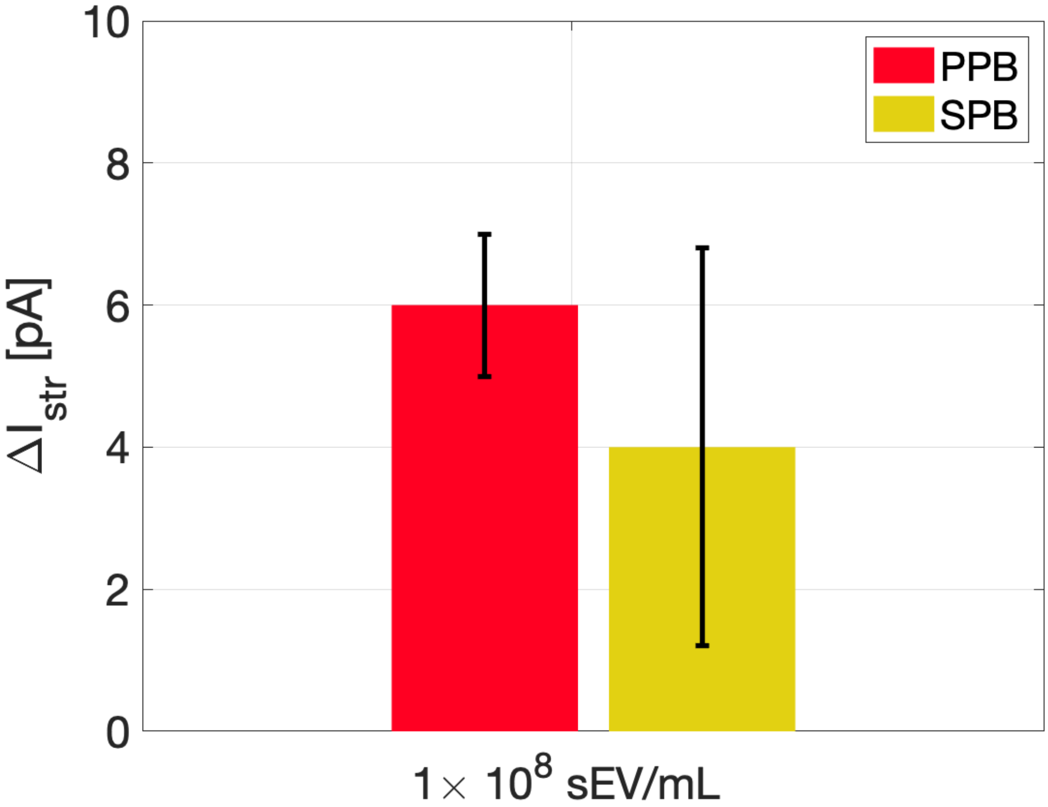

Figure S12 – Control measurements for SPB and PPB surfaces at the highest concentration of sEVs.

S15. Noise RMS and SNR comparison of the clean, PPB, and SPB functionalization strategies

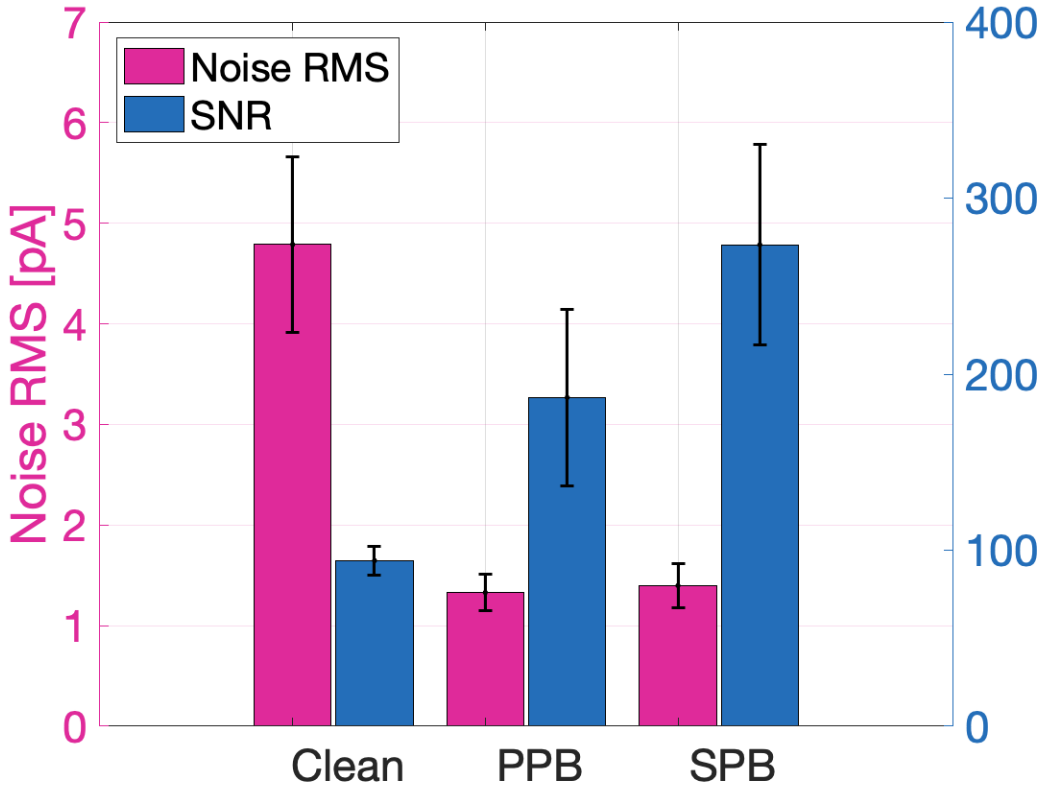

Figure S13 - The Signal to Noise Ratio (SNR) and noise RMS for the removable-top microchip in case of the clean, PPB, and SPB covered microchips at 150 kPa of upstream pressure. Data shown is from 3 technical repeats in all the cases.

S16. Slide angle measurement

To estimate the friction force between the liquid and the PPB/SPB surfaces, two flat silica coverslips were functionalized by PPB and SPB following the protocol stated in the manuscript. Thereafter a 4 µL droplet of 0.1x PBS was placed on the center of the surfaces. The slope of the surfaces was gradually increased until the droplets started to slide along the surfaces. An image was snapped at this movement and the friction force was estimated with data presented in Figure S14.

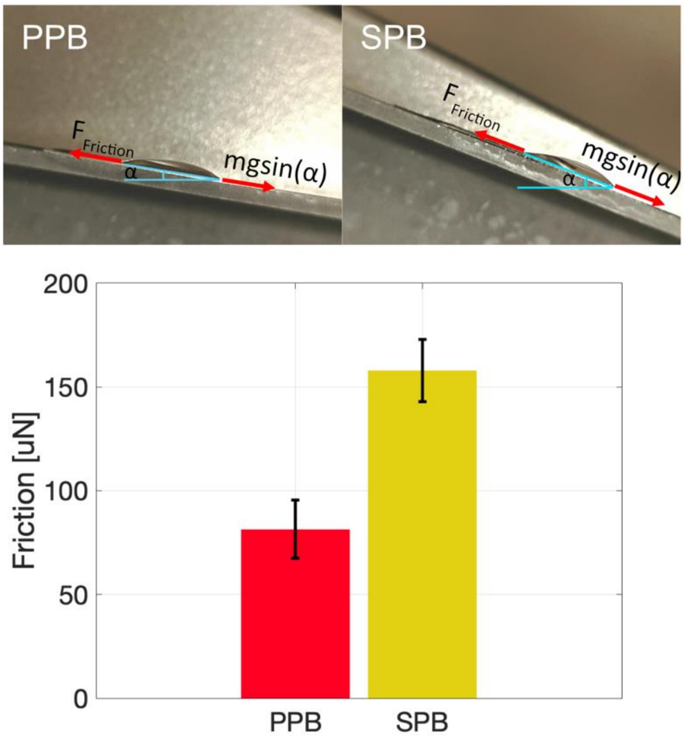

Figure S14 – Slide angle measurement and friction force estimation between the liquid and the surface on both PPB and SPB surfaces. Data shown is from 3 technical repeats.

References:

(1) Stiller, C.; Viktorsson, K.; Paz Gomero, E.; Hååg, P.; Arapi, V.; Kaminskyy, V. O.; Kamali, C.; De Petris, L.; Ekman, S.; Lewensohn, R. Detection of Tumor-Associated Membrane Receptors on Extracellular Vesicles from Non-Small Cell Lung Cancer Patients via Immuno-PCR. *Cancers (Basel).* **2021**, *13* (4), 922.

(2) Sahu, S. S.; Cavallaro, S.; Hååg, P.; Nagy, A.; Karlström, A. E.; Lewensohn, R.; Viktorsson, K.; Linnros, J.; Dev, A. Exploiting Electrostatic Interaction for Highly Sensitive Detection of Tumor-Derived Extracellular Vesicles by an Electrokinetic Sensor. *ACS Appl. Mater. Interfaces* **2021**.

(3) Adamczyk, Z.; Nattich, M.; Zaucha, M. Electrokinetics of Particle Covered Surfaces. *Curr. Opin. Colloid Interface Sci.* **2010**, *15* (3), 175–183.

(4) Cras, J. J.; Rowe-Taitt, C. A.; Nivens, D. A.; Ligler, F. S. Comparison of Chemical Cleaning Methods of Glass in Preparation for Silanization. *Biosens. Bioelectron.* **1999**, *14* (8–9), 683–688.

(5) Kirby, B. J. *Micro-and Nanoscale Fluid Mechanics: Transport in Microfluidic Devices*;

Cambridge university press, 2010.
